# Drug resistant glioblastoma stem cells exhibit enriched stemness signatures and share extracellular matrix overexpression

**DOI:** 10.1186/s12885-025-15163-z

**Published:** 2025-10-27

**Authors:** Lance L. Estabillo, Erlend Skaga, Skarphedinn Halldorsson, Einar O. Vik-Mo, Cecilie J. Sandberg

**Affiliations:** 1 Vilhelm Magnus Laboratory for Neurosurgical Research, Institute for Surgical Research and Department of Neurosurgery, Oslo University Hospital, Oslo, Norway; 2 Institute of Clinical Medicine, Faculty of Medicine, University of Oslo, Oslo, Norway

**Keywords:** Brain cancer, Glioblastoma, Glioblastoma stem cells, Drug sensitivity, Drug resistance, Extracellular matrix, Stemness, Resistance mechanisms, Individualized medicine

## Abstract

**Background:**

Understanding the complex molecular mechanisms driving drug resistance in glioblastoma (GBM) is crucial to develop effective therapeutic strategies. While prior studies have identified resistance mechanisms tied to specific drugs or pathways, a multi-modal molecular analysis of resistance across a range of drug classes is lacking.

**Methods:**

We identified highly drug-resistant (*n* = 5) and drug-sensitive (*n* = 4) cultures from a cohort of 32 patient-derived glioblastoma stem cell (GSC) cultures screened against a broad panel of ~ 500 anti-cancer drugs. To elucidate the key drivers of drug resistance, we performed integrative profiling of stemness, differentiation capacity, global gene expression, mutation profiles, and DNA methylation patterns between the two groups.

**Results:**

Despite heterogeneous gene expression profiles, drug-resistant GSCs showed consistent upregulation of ATP-binding cassette (ABC) drug efflux transporters, stemness, and extracellular matrix (ECM)-related genes. Compared to drug-sensitive GSCs, drug-resistant GSCs exhibited more pronounced stem-like properties and reduced differentiation capacity. Notably, genes linked to axonogenesis displayed significant CpG island hypomethylation in drug-resistant GSCs.

**Conclusions:**

This study suggests a pivotal role for GSC plasticity, stemness maintenance, and ECM-mediated drug evasion in GBM treatment resistance. Our findings highlight the adaptive and dynamic nature of resistance mechanisms in GSCs, emphasizing the need for comprehensive molecular insights to inform targeted therapeutic strategies in GBM.

**Supplementary Information:**

The online version contains supplementary material available at 10.1186/s12885-025-15163-z.

## Background

Glioblastoma (GBM) is the most common malignant primary brain tumor in adults [1]. The standard-of-care involves maximal safe tumor resection followed by radiotherapy and adjuvant temozolomide (TMZ) chemotherapy [1]. Despite this combined approach, the median survival for unselected patients remains poor at approximately 12 months [1]. The limited effectiveness of treatments is mainly due to the complex molecular and cellular tumor heterogeneity of GBM [2–4], which drives resistance to therapy [5].

Glioblastoma stem cells (GSCs), a subpopulation of tumor cells with stem-like properties, reside at the top of the tumor’s proliferative hierarchy and can recapitulate its entire cellular heterogeneity upon xenografting [4]. GSCs exhibit heightened resistance to both irradiation and chemotherapy, thereby sustaining therapy-resistant subpopulations, and are widely regarded as key drivers of treatment failure and tumor recurrence in GBM [4, 6, 7]. This resistance arises through both intrinsic mechanisms – where GSCs inherently resist therapy – and acquired mechanisms that develop over time in response to therapeutic pressures [8, 9].

Understanding the complex interplay of mechanisms driving therapy resistance in GSCs is crucial to develop more effective treatments for GBM. GSCs possess an enhanced capacity to undergo dedifferentiation and to recreate tumor heterogeneity following therapy [10, 11]. Their ability for epigenetic reprogramming, which may transition GSCs into a quiescent state, also underscore their plasticity to withstand therapy [11]. Several protective strategies in GBM have also been described, including the recruitment of ATP-binding cassette (ABC) drug efflux transporters [12], dysregulation of DNA repair systems [6, 13], and functional integration into neuronal circuits and the tumor microenvironment (TME) [14, 15]. Although several key resistance-driving features have been identified, most are derived from studies focused on individual drugs or specific molecular pathways [16–18]. A broad, integrative molecular characterization of the mechanisms underlying resistance across a wide panel of drug classes and mechanisms of action remains lacking.

GSCs derived from patient tumor biopsies preserve the parental tumor’s molecular makeup and tumorigenicity, providing a tumor model suitable for individualized drug sensitivity testing [19–21]. We have previously demonstrated substantial heterogeneity in drug sensitivity profiles among patient-derived glioblastoma stem cell (GSC) cultures exposed to ~ 500 anti-cancer drugs, revealing a continuum of highly drug-sensitive to highly drug-resistant phenotypes [22]. Here, we investigate molecular and cellular disparities between drug-resistant and drug-sensitive GSC cultures using a multi-modal approach, aiming to elucidate key drivers of intrinsic drug resistance in GSCs.

## Methods

### Patient-derived glioblastoma stem cell cultures

Biopsies were obtained from informed and consenting patients ongoing surgery for a glioblastoma at the Department of Neurosurgery, Oslo University Hospital, Norway. Neuropathological diagnostics were performed based on the version of WHO classifications at the time of biopsy and updated according to the WHO 2021 version [23]. The procurement of biopsies, biobanking, and subsequent analyses were approved by the Norwegian Regional Committee for Medical Research Ethics (REK 2016/1791 and 2017/166).

To capture cellular heterogeneity, the biopsies were harvested from several focal tumor regions. Patient-derived GSC cultures were established from both the tumor biopsies and ultrasonic aspirate. The cultures were maintained in serum-free, growth factor enhanced media and incubated at a density of 10^5^ cells/mL, as previously described [21]. The GSC cultures have been previously validated for stem cell properties [20, 22, 24, 25]. Differentiation was induced in growth factor free, serum supplemented media, as previously described [21, 26]. Patient characteristics are summarized in Additional file 1.

### Drug screening and drug sensitivity scoring

Drug sensitivity and resistance testing was performed using pre-drugged plates containing a panel of up to 527 anti-cancer drugs. The drug library comprised of both U.S. Food and Drug Administration and European Medicines Agency (FDA/EMA)-approved and investigational compounds, with various mechanisms of action, including conventional chemotherapies, kinase inhibitors, immunomodulatory drugs, epigenetic modifiers, hormone therapies, and apoptotic modulators [22]. GSCs were seeded at a density of 3000 cells/well and cell viability was measured using the Cell Titer-Glo Luminescent Assay after 72 h, as previously described [22]. Data were normalized to DMSO (negative control) and benzethonium chloride (positive control) wells.

Drug sensitivity was quantified using the Drug Sensitivity Score (DSS), a standardized parameter developed for functional investigation of druggable vulnerabilities in individual cancer samples ex vivo [27]. In brief, each drug was tested in a 5-point dose-escalating pattern covering a clinically relevant drug range. The resulting dose-response curve and curve fitting parameters were used to calculate the area of drug activity (10–100% inhibition relative to the positive and negative controls) to a single DSS measure.

### Western blot analysis

Protein was extracted using a lysis buffer (Invitrogen, M-PER™) supplemented with dithiothreitol (Sigma-Aldrich) and a phosphatase/protease inhibitor (Invitrogen), followed by homogenization using a QIAshredder column (Qiagen). Protein concentrations were quantified using a BCA assay kit (Invitrogen, Pierce™) according to the manufacturer’s instructions, and a microplate reader (Perkin Elmer, Victor™ X5).

Protein extracts were denatured and diluted with β-Mercaptoethanol (Sigma-Aldrich) and 4X LDS sample buffer (Invitrogen) to a final concentration of 1 µg/µL. Protein lysates (30 µg) were loaded into 4–12% Tris-Glycine gels (Invitrogen). Protein gels were blotted onto 0.45 μm PVDF membranes using a Trans Blot-Cell (BioRad) as described by the manufacturer. The membranes were blocked with 1X TBS/0.1% Tween-20, containing 5% non-fat dry milk (BioRad) and probed with primary antibodies diluted in 1X TBST either containing 5% BSA (Sigma-Aldrich) or 5% non-fat dry milk (BioRad). Secondary antibodies were HRP-conjugated anti-rabbit/mouse/rat IgGs. The complete list of antibodies is shown in Additional file 2. For stripping, the membrane was treated with a solution containing glycine (Sigma-Aldrich, 15 g/L), SDS (Sigma-Aldrich, 1 g/L), and 1% Tween-20 (Sigma-Aldrich), adjusted to pH 2.2, for a total of 1 h. Protein bands were visualized using SuperSignal™ West Femto Substrate (Invitrogen), imaged using the ChemiDoc™ Imaging System (BioRad), and quantified with Image Lab Software (BioRad). Protein band quantification was normalized to β-actin and, for differentiated samples, also to their undifferentiated counterparts. Differentiated samples were quantified relative to their undifferentiated counterparts, which were assigned a baseline value of 1.

### Mutation profiling

DNA from GSCs was isolated using the AllPrep DNA Micro Kit (Qiagen). Sequencing was performed in two batches using different exome capture kits: Agilent Human All Exon v5 (batch 1) and Twist Exome 2.0 plus Comprehensive Exome Spike (batch 2). DNA libraries were sequenced using paired-end reads on the Illumina HiSeq 4000 (batch 1) or NovaSeq 6000 (batch 2). Read alignment to the GRCh38 reference genome, together with sorting, duplicate marking, and somatic variant calling (tumor-only mode), was carried out with the Illumina Dragen platform (v4.2.4). To harmonize batches, variants were filtered with bedtools against genomic regions shared between both exome designs. Variants were annotated and classified with the Personal Cancer Genome Reporter v1.4.1 [28], using ClinVar, CIViC, CGI, and COSMIC. Clinical relevance was assigned according to the four-tier system [29]. Analyses focused on Tier I-III genes with allele frequency > 5%, and TCGA-defined significantly mutated GBM genes [3].

### Flow cytometry analysis

GSCs were enzymatically dissociated into single cells, passed through a 40 µm filter, and cultured at a density of 5 × 10^5^ cells/mL containing 10µM 5-ethynyl-2’-deoxyuridine (EdU) and incubated for 24 h. Flow samples were prepared using the Click-iT™ EdU Alexa Flour-488 Flow Cytometry Assay Kit (Invitrogen) according to the manufacturer’s instructions. Analysis was performed using a BD LSRFortessa™ cell analyzer (BD Biosciences) at the Flow Cytometry Core Facility, Oslo University Hospital. FlowJo™ software v10.10 was used for subsequent data analysis. Dead cells and doublets were excluded by gating.

### Nanopore sequencing and DNA methylation analysis

Genomic DNA was extracted using the QIAamp DNA Micro Kit (Qiagen), according to the manufacturer’s protocol. DNA purity and concentration were determined using a NanoDrop™ One Spectrophotometer and a Qubit™ Fluorometer (both Thermo Fisher Scientific). Native barcoding of genomic DNA was carried out using the Native Barcoding Kit 24 v14 (Oxford Nanopore Technologies), according to the manufacturer’s protocol. The final DNA library was purified with AMPure XP beads (Beckman Coulter) and pools of four samples were loaded onto PromethION R10.4.1 flow cells. High-throughput nanopore sequencing was performed using the PromethION 24 sequencer (Oxford Nanopore Technologies) for a total of 72 h. A minimum whole-genome sequence depth of 5 was established pre-analysis. Modified bases (5mC) were identified using the Dorado basecaller (v0.7.3) (https://github.com/nanoporetech/dorado), with binary alignment map file manipulation for downstream analyses done using Modkit (v0.3.1) (https://github.com/nanoporetech/modkit), both from Oxford Nanopore.

CpG islands were identified using a reference obtained from UCSC Human Genome Assembly (https://hgdownload.soe.ucsc.edu/goldenPath/hg38/database/cpgIslandExt.txt.gz). Methylation percentages of individual CpG islands were calculated by averaging the methylation levels of all CpG sites within each island, in conjunction with Modkit. Differential methylation analysis between drug-resistant and drug-sensitive groups was performed using independent two-sample t-tests in R (v4.2.1), with CpG islands considered differentially methylated at *p* < 0.05. CpG islands linked to gene expression changes were identified by comparing methylation levels with the expression levels of the associated gene; an inverse correlation was indicative of a regulatory relationship. The source code used to reproduce all the analyses is available at request. MGMT methylation was calculated as the average methylation of all 98 CpG sites within its promoter (chr10:129,466,685 − 129,467,446, hg38). GSC cultures with an average methylation of > 22% were considered MGMT methylated according to the established optimal methylation cutoff using nanopore sequencing [30]. Visualization of MGMT methylation patterns was done using methylartist (v1.3.0).

### RNA-sequencing and expression data analysis

RNA was extracted using the RNeasy Micro Kit (Qiagen), and evaluated through the NanoDrop™ One Spectrophotometer (Thermo Fisher Scientific) and the Agilent 2100 Bioanalyzer (Agilent Technologies). RNA sequencing was performed by the Genomics and Bioinformatics Core Facility at Oslo University Hospital. In brief, library preparation of high-quality RNA (RIN > 8) was performed using the TruSeq mRNA (Illumina) protocol, and the samples were sequenced on the Illumina system using paired-end 2 × 75 bp reads. Further processing was conducted using the Tuxedo pipeline (Cufflinks Assembly and DE (BaseSpace Workflow), Isis (Analysis Software), STAR (Aligner), Isaac Variant Caller, VEDTools, Cufflinks, and BLAST). FPKM (fragments per kilobase of transcript per million fragments mapped) data per sample were extracted and the raw count matrix was further analyzed in R. Normalization and differential gene expression (DGE) analysis were done with DESeq2 (v1.42.1). Gene ontology enrichment analysis and principal component analysis (PCA) were performed using clusterProfiler (v4.10.1) and mixOmics (v6.26.0) in R.

Classification into proneural (PN) and mesenchymal (MES) subtypes was performed using two predefined gene sets: (i) a GSC-specific panel [22] and (ii) a TCGA tissue-derived panel [31]. For each panel, gene expression counts were normalized using variance-stabilizing transformation in DESeq2 (v1.42.1). Single-sample gene set enrichment analysis (ssGSEA) was conducted using the GSVA package (v1.50.5) to generate enrichment scores for each subtype-specific gene signature. PCA was applied to the normalized expression profiles to visualize subtype clustering.

### Statistical considerations

All two-group comparisons between drug-resistant and drug-sensitive samples (e.g., protein marker levels, number of drugs with DSS > 10, enrichment set scores, cumulative expression levels, MGMT promoter methylation, and TMZ sensitivity) were performed using unpaired two-tailed t-tests with Welch’s correction. Unsupervised hierarchical clustering and heat maps were generated using the ComplexHeatmap (v2.18.0) and pheatmap (v1.0.12) packages in R, with standard Euclidean distance and Ward.D2 linkage. Heatmap visualization of gene expression and methylation data were scaled by subtracting the row mean and dividing by the row standard deviation, centering each row around zero with a standard deviation of one. DSS values were normalized to the average DSS of all screened GSC cultures per drug for unsupervised hierarchical clustering. Multiple testing correction for differential gene expression analysis was performed using the Benjamini-Hochberg false discovery rate method. An independent two-sample t-test of methylation levels was used to identify differentially methylated CpG islands, considering p-values < 0.05 as statistically significant.

## Results

### GSC cultures separate into drug-sensitive and drug-resistant groups

**Fig. 1:**
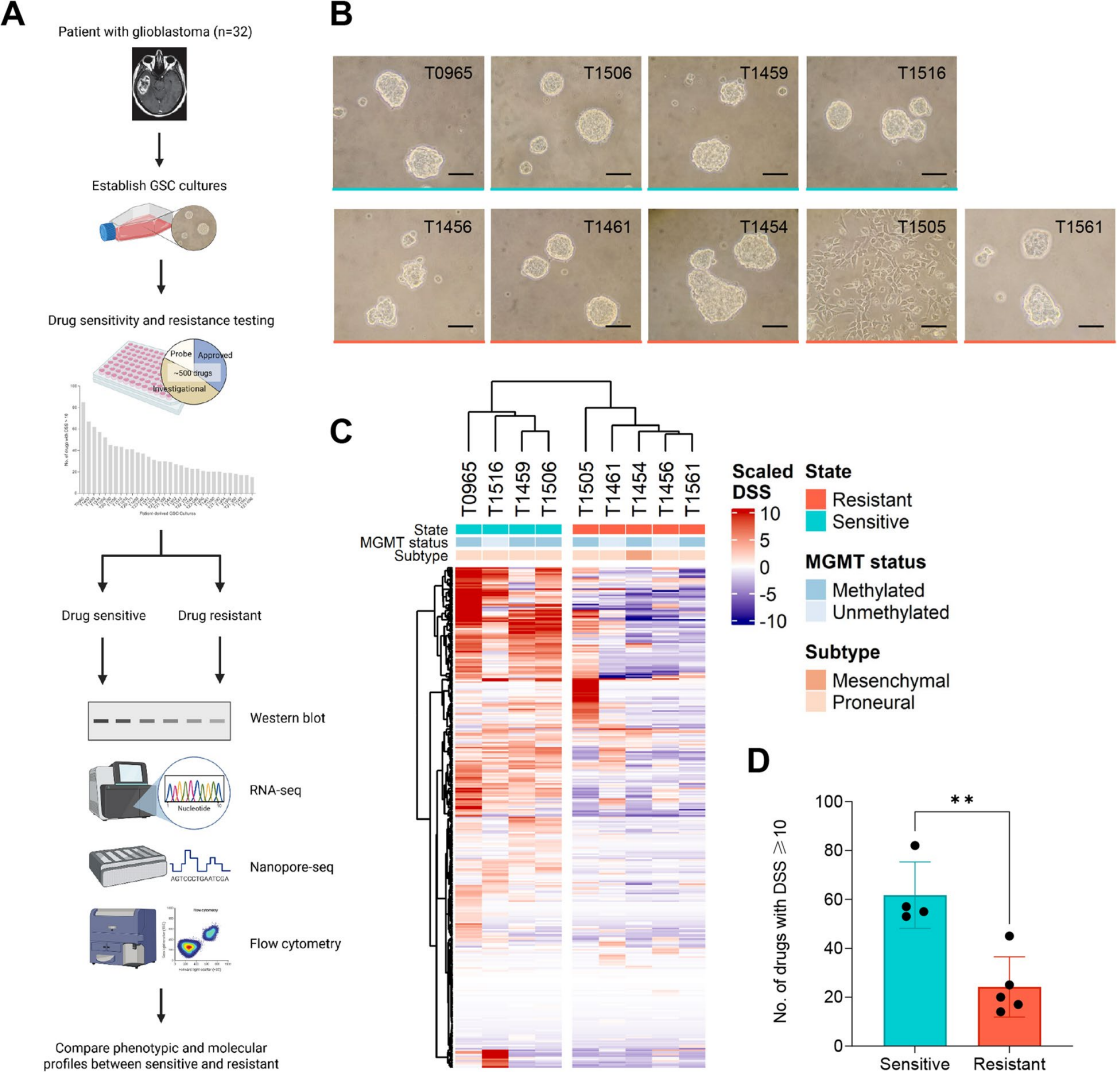
GSC cultures separate into drug-sensitive and drug-resistant groups. (**A**) Overview of the study design. Created in BioRender (2025) https://BioRender.com/7r8xuu15nkr3tf. (**B**) Images showing the phenotypes of the GSC cultures (*n* = 9). Scale bar: 100 μm. (**C**) Unsupervised hierarchical clustering separating the highly drug-sensitive and highly drug-resistant GSC cultures into two distinct groups. Clustering was based on the DSS scores of each drug normalized against the average DSS score across all screened GBMs. (**D**) Number of drugs eliciting a response of DSS ≥ 10 in each cluster. **p < 0.01

From a cohort of 32 patient-derived GSC cultures screened against a broad panel of up to 527 anti-cancer drugs per sample [22], we identified representative GSC cultures from the extremes of the drug response spectrum to investigate molecular features associated with highly drug-resistant and drug-sensitive phenotypes. An overview of the study design is shown in Fig. 1A.

Individual drug responses were quantified using the Drug Sensitivity Score (DSS), which integrates the area under the estimated dose-response curve to assess sensitivity [27]. Based on previous studies [20, 22], a DSS ≥ 10 was used to define a moderate to high drug response. Each of the 32 GSC cultures were ranked according to the total number of drugs showing DSS ≥ 10, enabling classification of drug sensitivity across the cohort (Additional file 3 A, Additional file 4).

We identified four GSC cultures as highly drug-sensitive (T0965, T1459, T1516, T1506), and five as highly drug-resistant (T1456, T1561, T1505, T1454, T1461). Eight GSC cultures proliferated as free-floating tumor spheres, while one (T1505) proliferated adherently in culture (Fig. 1B). There was no significant difference in proliferation rate between the two groups and all cultures maintained their morphology through serial passages (Additional file 5).

Unsupervised hierarchical clustering of drug response profiles, restricted to compounds tested across all cultures (*n* = 329), separated the GSC cultures into two distinct and robust clusters (Fig. 1C, Additional file 3B, Additional file 4). Both MGMT methylated and MGMT unmethylated tumors were represented in each cluster. Subtype classification using two independent gene panels: (i) GSC-specific and (ii) TCGA tissue-derived gene set, identified eight GSC cultures as PN and one as MES, with the mesenchymal culture falling into the drug-resistant group (Additional file 6 A-B). Notably, analysis of enrichment scores revealed that drug-resistant cultures had significantly higher MES scores compared to drug-sensitive cultures (i: *p* = 0.039, ii: *p* = 0.009), whereas drug-sensitive cultures displayed higher PN scores (i: *p* = 0.047, ii: *p* = 0.012) (Additional file 6 C). The two clusters differed significantly in the number of drugs eliciting a response of DSS ≥ 10, with drug-sensitive GSC cultures responding to a higher number of drugs (Fig. 1D, *p* = 0.0034).

### Drug-resistant GSCs maintain stemness markers and show impaired differentiation capacity

**Fig. 2:**
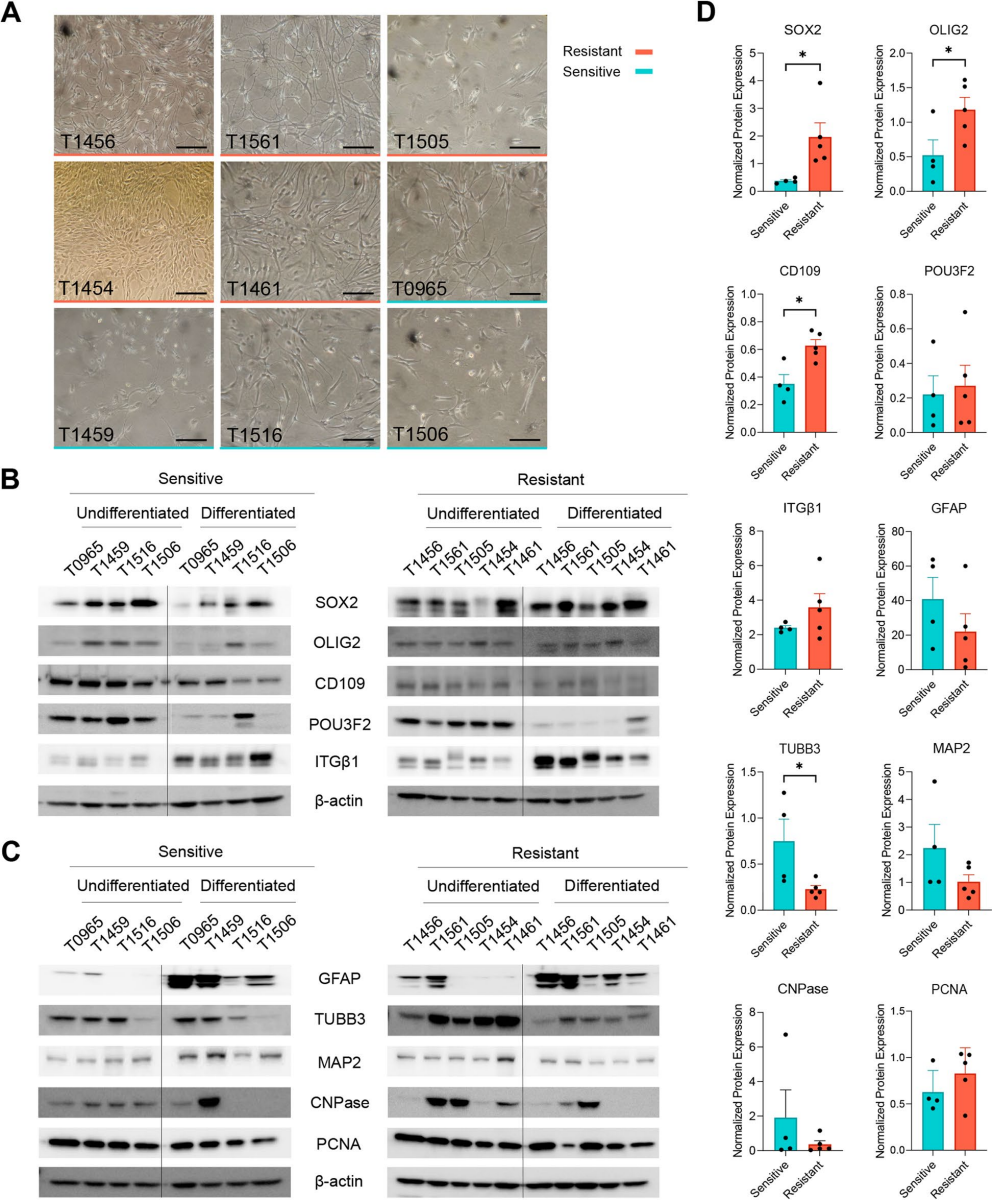
Drug-resistant GSCs maintain stemness markers and show impaired differentiation capacity. (**A**) Images of the GSC cultures upon exposure to differentiating conditions. Scale bar: 50 μm. (**B**) Protein expressions of the stemness markers SOX2, OLIG2, CD109, POU3F2, ITGβ1, and (**C**) Protein expressions of the mature neural cell markers GFAP (astrocytes), TUBB3 & MAP2 (neurons), and CNPase (oligodendrocytes), alongside the proliferation marker PCNA in undifferentiated and differentiated, drug-sensitive (left) and drug-resistant (right) GSC cultures. Full-length blots are presented in Additional file 7. (**D**) Quantification of protein expression for each molecular marker. Expression is normalized to β-actin and calculated relative to their corresponding undifferentiated control samples. Individual points represent each GSC culture with error bars as means with SD. *p < 0.05

For functional characterization, we first explored the differentiation capacities of drug-resistant and drug-sensitive GSC cultures into the three main neural lineages. Upon differentiation, all the GSC cultures proliferated adherently and showed heterogenous morphologies with varied arborizations (Fig. 2A).

Compared to the drug-sensitive GSCs, drug-resistant GSCs showed a significant increase in SOX2 (*p* = 0.035), OLIG2 (*p* = 0.049), and CD109 (*p* = 0.016) protein levels under differentiating conditions (Fig. 2B and D). Conversely, drug-resistant GSCs exhibited lower TUBB3 (*p* = 0.046) protein levels than drug-sensitive GSCs after differentiation (Fig. 2C and D).

Both groups demonstrated a higher propensity for astrocyte differentiation compared to neuronal and oligodendrocyte differentiation, reflected by the substantial increase in GFAP protein post-differentiation, relative to their undifferentiated counterparts (Fig. 2C and D). PCNA protein levels remained comparable between groups and differentiation states, indicating no significant difference in proliferation following differentiation (Fig. 2C and D). Taken together, these results suggest that drug-resistant GSCs sustain higher stemness marker expression post-differentiation, and have reduced capacity for neuronal differentiation compared to drug-sensitive GSCs.

### Drug-resistant GSCs show more diverse gene expression profiles, with ABC transporter and stemness gene upregulation

To further characterize the molecular features of intrinsic GSC resistance, we investigated differences in mutations and global gene expression profiles between drug-resistant and drug-sensitive GSC cultures.

**Fig. 3:**
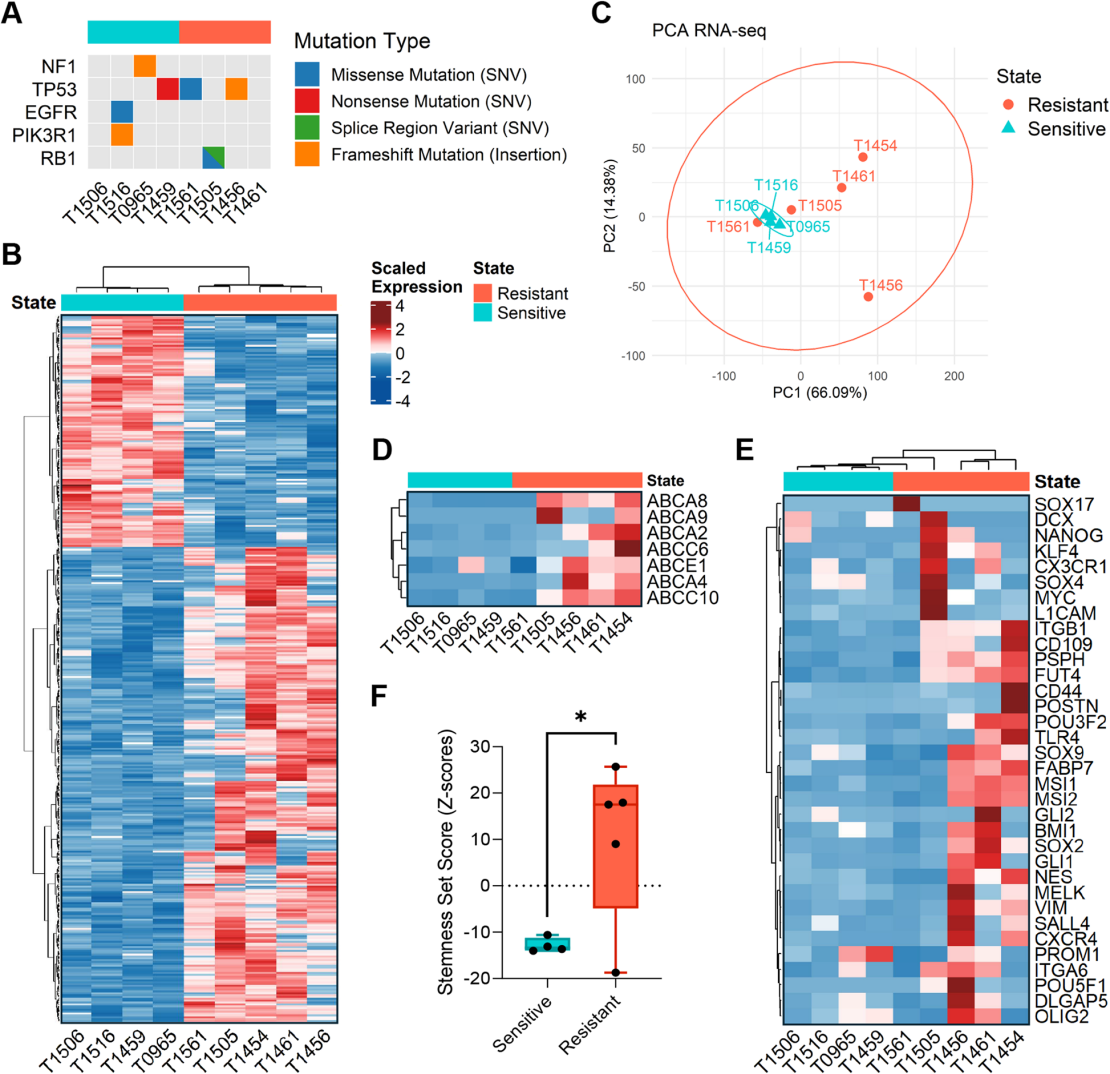
Drug-resistant GSCs show more diverse gene expression profiles, with ABC transporter and stemness gene upregulation. (**A**) Mutation profiles of drug-resistant and drug-sensitive GSC cultures, restricted to TCGA-defined significantly mutated GBM genes with an allele frequency > 5%. Genes with two mutations are split diagonally, with each half indicating a distinct mutation type. (**B**) Heatmap of the differentially expressed genes (*n* = 384) between both groups. (**C**) Principal component analysis based on the global gene expression profiles of drug-resistant and drug-sensitive GSC cultures. (**D**) Heatmap summarizing the gene expression levels of ABC drug efflux pumps (*n* = 7) between the two groups. (**E**) Unsupervised hierarchical clustering of the expression of stemness genes (*n* = 34) in both groups. (**F**) Stemness set scores for the drug-resistant (10.3) and drug-sensitive (−12.8) GSCs, calculated as the average expression of all stemness genes using their rank-normalized Z-scores

We did not identify any Tier I or Tier II mutations (variants with strong or potential clinical significance) in the GSC cultures. In contrast, 45 mutated genes were classified as Tier III variants (variants of uncertain significance, Fig. 3A, Additional files 8 and 9). Of these, only five genes (EGFR, NF1, PIK3R1, RB1, TP53) are reported by TCGA to be significantly altered in GBM [3], and with one exception, these mutations were found in single GSC cultures only. Thus, no consistent mutational pattern could be linked to either the drug-sensitive or drug-resistant phenotypes.

Differential gene expression (DGE) analysis identified 384 genes that were significantly, differentially expressed between the two groups (adjusted *p* < 0.05; Fig. 3A, Additional file 10). Principal component analysis (PCA) revealed dense clustering among drug-sensitive GSC cultures, indicating a marked similarity in transcriptional patterns, whereas drug-resistant GSC cultures showed a scattered distribution, suggesting greater heterogeneity in gene expression profiles (Fig. 3B). Notably, several ABC drug transporter genes (*n* = 7) were differentially expressed between groups. Despite one outlier (T1561), drug-resistant GSCs exhibited higher expression of these genes, with ABCA4 (*p* = 0.009), ABCE1 (*p* = 0.008), and ABCC6 (*p* = 0.002) showing the most significant differences (Fig. 3C).

We next examined the expression of key stemness-associated genes (*n* = 34) across the cultures [32, 33]. With the exception of T1561, the drug-resistant GSCs displayed an overall upregulation of stemness genes compared to drug-sensitive GSCs (Fig. 3D). Considerable heterogeneity in stemness gene expression profiles was observed across individual samples, underscoring the molecular variability within GBM (Fig. 3D).

To quantify overall stemness within each group, a stemness set score was calculated based on the mean of rank-normalized expression values (Z-scores). Drug-resistant GSCs had a significantly higher stemness set score (10.3) compared to drug-sensitive GSCs (−12.8; *p* = 0.034) (Fig. 3E). Together, increased stemness expression was observed among drug-resistant GSCs, suggesting that intrinsic drug resistance is associated with both transcriptional diversity and a more stem-like state.

### Drug-resistant GSCs show enrichment of collagen and extracellular matrix pathways

**Fig. 4:**
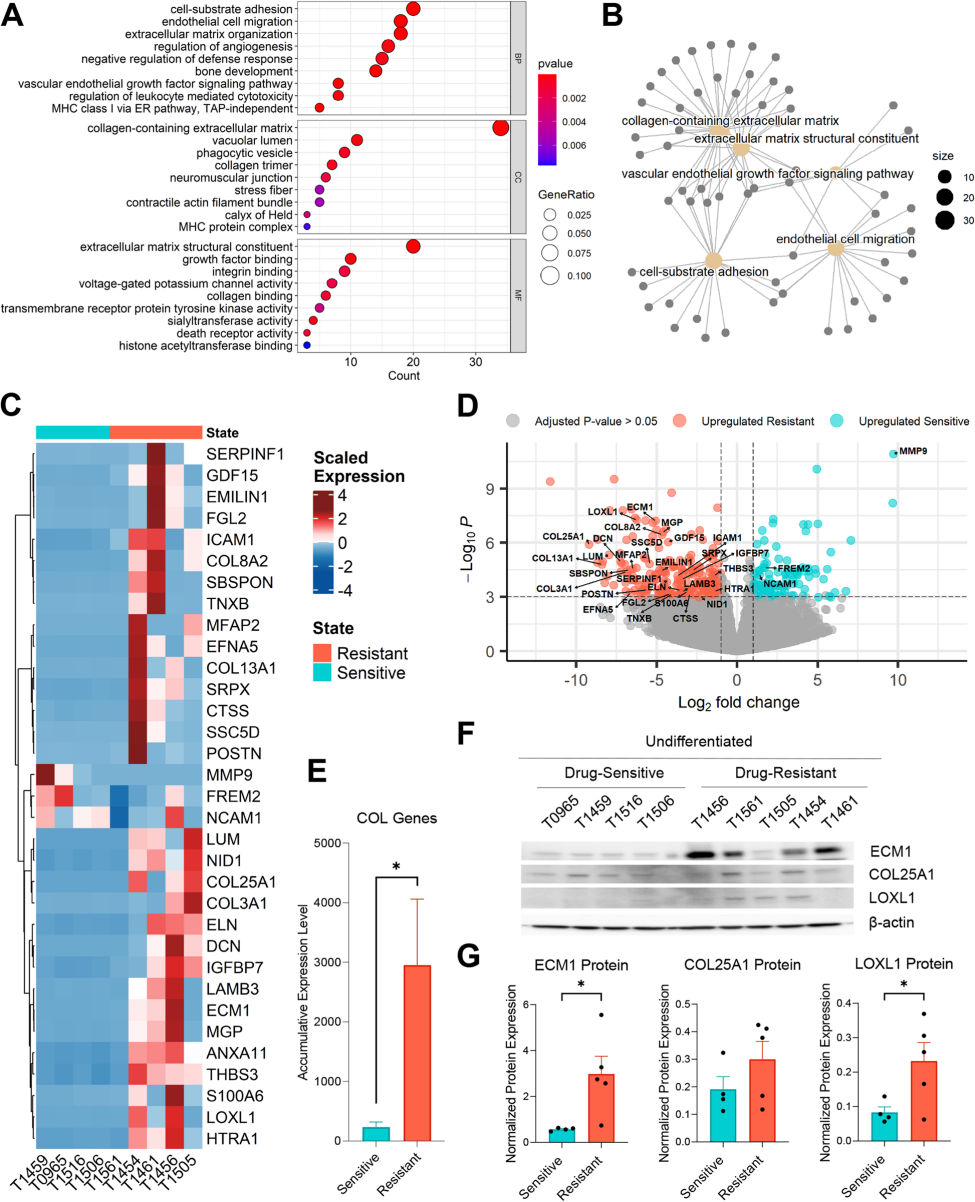
Drug-resistant GSCs show enrichment of collagen and extracellular matrix pathways. (**A**) Dot plot representing the gene ontology (GO) terms most highly enriched associated with the differentially expressed genes between drug-resistant and drug-sensitive GSCs. BP = biological process, CC = cellular component, MF = molecular function. (**B**) Network of the top five, most significantly enriched GO terms between the two groups, with their associated genes as gray nodes. (**C**) Heatmap of the expression levels of each gene (*n* = 34) within the extracellular matrix (ECM) and collagen (COL) GO terms. (**D**) Volcano plot of the differentially expressed genes between the two groups. Genes with a log2 fold change > 1 and an adjusted p-value < 0.001 are highlighted in blue and red, with the ECM and COL-associated genes annotated. (**E**) The average expression of the differentially expressed COL genes (*n* = 15) in each group. Error bars indicate means with SD. (**F**) Protein expression of the ECM and COL genes ECM1, COL25A1, and LOXL1 in drug-sensitive and drug-resistant GSC cultures. Full-length blots are presented in Additional file 7. (**G**) Quantification of protein expression for each gene. Expression is normalized to β-actin. Individual points represent each GSC culture with error bars indicating means with SD. *p < 0.05

To identify biological processes associated with drug-resistance, we further performed over-representation analysis (ORA) on the differentially expressed genes (*n* = 384) based on gene ontology (GO) annotations. The most significantly enriched GO terms included collagen (COL)-containing extracellular matrix (ECM) (padj = 2.0 × 10^−14^) and ECM structural constituent (padj = 8.6 × 10^−12^) (Fig. 4A). Together with these, the top five most significantly enriched terms also included endothelial cell migration (padj = 1.4 × 10^−6^), vascular endothelial growth factor (padj = 1.8 × 10^−6^), and cell-substrate adhesion (padj = 2.4 × 10^−6^) (Fig. 4B). Gene set enrichment analysis (GSEA) was also performed, showing similar results (Additional file 11).

The majority of the ECM- and collagen-associated genes (*n* = 30/34) showed higher expression in drug-resistant GSCs (Fig. 4C and D, Additional file 12 A). Notably, these genes were primarily related to ECM remodeling, formation, and structural integrity. Substantial heterogeneity in ECM gene expression profiles was also evident across individual GSC cultures (Fig. 4C). Further, a higher expression of collagen genes (*n* = 15) was observed in drug-resistant compared to drug-sensitive GSCs, with *COL3A1* (padj = 0.006), *COL25A1* (padj = 0.0005), *COL13A1* (padj = 0.004), and *COL8A2* (padj = 0.0002) among the most significantly upregulated (Fig. 4E, Additional file 12B). In line with gene expression, protein levels of ECM-associated genes, *ECM1* (*p* = 0.029) and *LOXL1* (*p* = 0.049) were significantly upregulated in drug-resistant GSCs (Fig. 4F-I). Thus, drug-resistant GSCs possess heterogenous transcriptional profiles, but collectively exhibit increased expression of ECM- and collagen-associated genes.

### Drug-resistant and drug-sensitive GSCs show distinct CpG island methylation patterns linked to axonogenesis

Correlation between methylation in CpG islands and drug resistance have been found in several cancers, both prior to chemotherapy and acquired during treatment [34]. To identify DNA methylation patterns related to intrinsic drug resistance, we compared global 5-methylcytosine (5mC) patterns between drug-resistant and drug-sensitive GSC cultures.

**Fig. 5:**
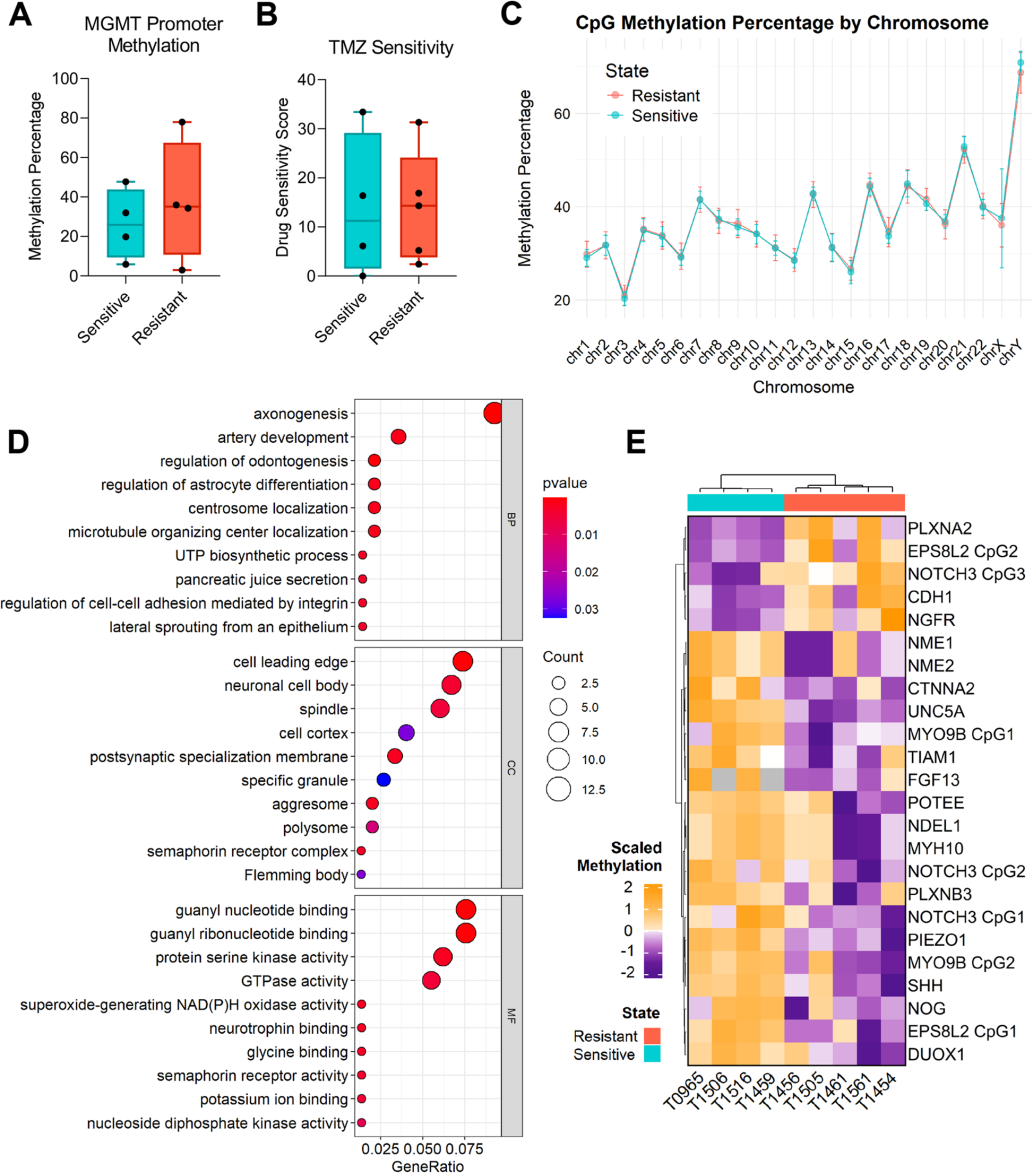
Drug-resistant and drug-sensitive GSCs show distinct CpG island methylation patterns linked to axonogenesis. (**A**) Average MGMT promoter methylation levels, and (**B**) TMZ drug sensitivity scores (DSS) between drug-resistant and drug-sensitive GSC cultures. Singular points represent each individual GSC culture. (**C**) Global CpG island (n = 27941) mean methylation levels, partitioned by chromosome in drug-resistant and drug-sensitive GSCs. Error bars indicate means with SD. (**D**) Dot plot representing the gene ontology (GO) terms most highly enriched among genes linked with the differentially methylated CpG islands. BP = biological process, CC = cellular component, MF = molecular function. (**E**) Unsupervised hierarchical clustering of the CpG island methylation levels for each gene (*n*=24) associated with the axonogenesis and cell leading edge GO terms in both groups

MGMT promoter methylation did not provide clear separation between drug-resistant and drug-sensitive GSCs (*p* = 0.549) (Fig. 5 A, Additional file 13 A). Similarly, TMZ sensitivity data [20] showed no discernable differences between groups (*p* = 0.996) (Fig. 5B). Comparison of mean global CpG island methylation levels (*n* = 27941) also revealed no significant differences between drug-resistant (35.28%) and drug-sensitive (35.08%) GSCs (*p* = 0.864) (Fig. 5C, Additional file 13B). However, a total of 675 CpG islands were significantly, differentially methylated between the two groups (*p* ≤ 0.05), of which 172 correlated with changes in gene expression (Additional file 13 C). Gene ontology analysis of these genes revealed enrichment in axonogenesis (padj = 2.9 × 10^−5^) and cell leading edge pathways (padj = 3.5 × 10^−4^) (Fig. 5D).

Analysis of genes within these pathways (*n* = 20) showed higher CpG methylation in drug-sensitive GSCs, indicating silencing of these genes in this group compared to drug-resistant GSCs (Fig. 5E, Additional file 14 A). The most significant differential methylations were observed for EPS8L2 (*p* = 0.005), PIEZO1 (*p* = 0.005), FGF13 (*p* = 0.0006), and UNC5A (*p* = 0.0005) (Additional file 14 A). An inverse relationship between DNA methylation and gene expression was also evident, with higher methylation correlating with reduced gene expression (Additional file 14B). Overall, these findings suggest that epigenetic regulation of genes involved in axonogenesis and cell motility, showing hypermethylation in drug-sensitive GSCs and hypomethylation in drug resistant GSCs, is associated with intrinsic drug resistance.

## Discussion

Using various profiling techniques, we have compared cellular and molecular properties between drug-sensitive and drug-resistant patient-derived GSC cultures to identify the mechanisms underlying intrinsic drug resistance in glioblastoma. Our results revealed three key insights; (i) drug-resistant GSCs display enhanced stem-like properties compared to drug-sensitive GSCs, (ii) drug-resistant GSCs exhibit greater heterogeneity in global gene expression, yet share an enrichment of ABC drug efflux pumps and ECM-associated genes, and (iii) differentially methylated CpG islands in drug-resistant GSCs exhibit hypomethylation of genes associated with axonogenesis and cell leading edge dynamics.

First, we found that highly drug-resistant GSCs displayed reduced differentiation capacity into the neuronal lineage compared to drug-sensitive GSCs. This was also accompanied by either sustained or elevated protein expression of stemness markers following differentiation, and a higher expression of various stemness-related genes compared to drug-sensitive GSCs. Specifically, we found that drug-resistant GSCs expressed significantly higher CD109 protein levels compared to drug-sensitive GSCs. This aligns with recent findings showing that CD109 is essential for maintaining stemness, and that its genetic targeting can reprogram resistant GSCs into a more chemo-sensitive state [35]. In addition, protein levels of SOX2 and OLIG2 were significantly increased in drug-resistant GSCs following differentiation. These transcription factors are key regulators involved in reprogramming differentiated GBM cells into stem-like tumor cells [36]. Both proteins have also been shown to enhance tumorigenicity, cellular plasticity, and drug resistance in GBM [37, 38]. These protein expression changes suggest that highly drug resistant GSCs respond to in vitro differentiation, at least in part, by reinforcing stemness – resembling a dedifferentiation process. Together, this differentiation-induced upregulation of stemness, increased expression of stemness-related genes, combined with a reduced differentiation capacity, supports a link between a heightened stem-like state and resistance to targeted drug therapies in GBM.

In contrast to the highly similar expression patterns in drug-sensitive GSCs, we found that drug-resistant GSCs exhibit significantly greater heterogeneity in their global gene expression profiles. Despite this heterogeneity, they collectively showed significantly higher MES transcriptional activity compared to drug-sensitive GSCs, while drug-sensitive GSCs exhibited higher PN signatures. This pattern was consistent across two independent gene panels, despite the cohort being predominantly classified as proneural. In GBM, the acquisition of therapy resistance and a more aggressive tumor phenotype under selective pressure has been shown to be driven by a proneural-to-mesenchymal transition [39]. The higher MES enrichment in drug-resistant GSCs therefore indicates that broad-spectrum drug resistance is similarly associated with an increased mesenchymal-like transcriptional state in GSCs.

Drug-resistant GSCs also showed elevated expression of ABC drug efflux pumps, as well as ECM- and COL-associated genes. In line with recent studies, various ABC drug pumps in GBM, specifically ABCB4 and ABCA13, have been shown to confer resistance to TMZ [40, 41]. Studies have also highlighted the impact of ECM stiffness in inducing hypoxia, reducing interstitial transport, and impeding vascular access to tumors, with collagen abundance correlating with increased tumor stiffness [42–45]. Consistent with our findings, elevated tumor and ECM stiffness have been linked to reduced survival in GBM [46], and therapeutic resistance across multiple cancer types [47–49]. This elevated expression of ECM and ABC drug pump genes in highly drug-resistant GSCs suggests the induction of an ECM barrier that both hinders interactions with drug agents and promotes drug efflux. This dense ECM may also reinforce stemness to support proliferation under hypoxic conditions, linking ECM enrichment and heightened stemness in drug-resistant GSCs. Collectively, the upregulation of ECM-associated genes and ABC drug transporters in drug-resistant GSCs, despite heterogenous gene expression profiles, points to their shared contribution to intrinsic drug resistance.

While the drug-resistant GSCs overall exhibited elevated expression of ABC transporter and stemness-associated genes relative to drug-sensitive GSCs, one drug-resistant sample (T1561) demonstrated a distinct transcriptional profile. This sample showed comparatively lower expression of these gene sets than other drug-resistant GSCs, which underscores the transcriptional heterogeneity observed within the drug-resistant group and suggests the presence of alternative resistance mechanisms that merit deeper exploration. Nonetheless, the overarching pattern across the broader cohort supports a molecular profile characterized by elevated drug efflux and enhanced stem-like properties as key drivers of drug resistance in GBM.

We further found that intrinsic resistance to targeted drugs is independent of MGMT methylation status, with no differences in mean global DNA methylation between drug-sensitive and drug-resistant GSCs. This diverges from previous studies linking high global DNA methylation to improved survival and radiotherapy sensitivity in GBM [50]. However, further in-depth analysis of epigenetic patterns within CpG islands identified enrichment of hypomethylated genes related to axonogenesis and cell leading edge dynamics among drug-resistant GSCs. Recent studies have linked axonogenesis to therapeutic resistance through neurotrophin-mediated nerve-cancer crosstalk, enhancing adaptive plasticity, stemness maintenance, and intratumoral sympathetic activation as a stress response to increase cell resistance [51–55]. Integration of GBM cells into brain-wide neuronal circuits have also been demonstrated, showing diverse connectivity [15]. Additionally, GBMs with hypomethylated CpGs and upregulated synaptic integration genes have also been found to exhibit increased therapeutic resistance and harbor more stem-like cells [56], consistent with our previous findings. Our results therefore indicate that drug-resistant GSCs promote axonogenesis to facilitate nerve-cancer crosstalk, inducing stress responses, plasticity, and therapeutic resistance. Together, this reveals axonogenesis as a key driver of intrinsic drug resistance in GBM through epigenetically regulated mechanisms.

Our findings provide new insight on the multifaceted molecular nature of drug resistance in GBM, revealing the convergence of intrinsic cellular plasticity, transcriptional heterogeneity, ECM dynamics, and epigenetic regulation in supporting a therapy-resistant state. Unlike prior studies focusing on single drugs or specific pathways, our analysis encompasses a diverse panel of drugs, offering a broader perspective on shared and drug class-independent resistance mechanisms in GBM. Our findings also identify actionable vulnerabilities and reveal genes enriched in drug-resistant GSCs, associated with stemness (e.g. SOX2), ECM remodeling (e.g. LOXL1), and axonogenesis (e.g. PIEZO1, SHH). Multiple studies across various cancers have already shown enhanced treatment efficacy by targeting ECM components [57, 58], ABC drug efflux pumps [59, 60], stemness-related pathways such as Notch, Wnt, and Hedgehog [61], as well as epigenetic neural remodeling [52, 62], potentially informing novel therapies to overcome GBM drug resistance.

We also found data supporting our results in recent single-cell and spatial transcriptomics studies, showing that the molecular signatures of drug-resistant GSCs are spatially localized within distinct tumor niches. More specifically, these studies show that ECM and stemness marker expression are consistently elevated at the leading edge and perivascular regions in GBM, which harbor brain tumor-initiating cells [63, 64]. This expression is highest in mesenchymal-like cells, consistent with elevated MES signatures in drug-resistant GSCs, and myelinating cells at the leading edge [63, 64]. Similarly, spatial transcriptomics links matrisome remodeling, stemness, and collagen-binding signatures to the tumor periphery [65, 66], and axonogenesis-related genes to invasive niches harboring radial glial-like cells [67], implicating ECM remodeling and glioma-neuron network formation at the invasive GBM edge. Together, this localized convergence of drug-resistant GSC signatures at distinct tumor niches highlights region-specific vulnerabilities that may be therapeutically exploited. Additionally, our results reveal significant intertumoral heterogeneity in ECM- and stemness-related gene expression profiles among drug-resistant GSCs. This indicates that distinct gene combinations are selectively employed by individual tumors to support drug resistance, deeming it necessary to address diverse resistance profiles for personalized medicine approaches.

Since the identification of tumor cells exhibiting stem-like characteristics in GBM, patient-derived GSCs have emerged as a superior in vitro model compared to traditional serum-cultured cell lines [19, 21]. By preserving the molecular makeup of the parental tumor, the GSC model represents an individualized platform that captures clinical GBM heterogeneity, enabling robust molecular analyses. However, we acknowledge the inherent limitations of the GSC culture system; as important aspects of in vivo GBM biology, including the blood-brain barrier, immune interactions, and the full complexity of the tumor microenvironment, are not addressed. While our study provides valuable insights, our results are based on a limited sample size at both extremes of the drug response spectrum, which may limit generalizability. Larger patient cohorts would help improve the transferability of our study and better capture population variability. Three dimensional models, such as tumor organoids, have also emerged as promising platforms offering valuable opportunities moving forward. These systems can recapitulate the in vivo microenvironmental architecture and multicellular interactions governing plasticity- and ECM-driven dynamics, which can advance clinical translation of our findings. However, their clinical predictive value still remains uncertain.

## Conclusion

In summary, our integrative molecular profiling of patient-derived GSC cultures revealed that drug-resistant GSCs exhibit more pronounced stem-like properties compared to drug-sensitive GSCs. Although global gene expression profiles were heterogenous, drug-resistant GSCs exhibited consistent upregulation of ABC drug efflux transporters, stemness-associated genes, and ECM- and cell adhesion-related genes. In addition, hypomethylation of genes linked to axonogenesis distinguished the drug-resistant group. These findings highlight a critical role of GSC plasticity, stemness maintenance, and ECM-mediated drug evasion in driving broad-spectrum treatment resistance in GBM. Understanding these mechanisms is crucial for developing tailored therapies and advancing ex vivo drug screening strategies aimed at overcoming GBM drug resistance.

## Supplementary Information

Additional file 1. *Patient characteristics*. Clinical and demographic information of the patients from whom all GSC cultures were obtained.

Additional file 2. *Primary and secondary antibodies.* The complete list of the primary and secondary antibodies used in the study, with their host species and working dilutions.

Additional file 3. *Clustering of GSCs according to drug sensitivity.* A) Ranking from most drug-sensitive to most drug-resistant of 32 patient-derived GSC cultures screened against ~up to 527 anti-cancer drugs. The GSCs are ranked based on the number of drugs with DSS ≥ 10. Only drugs tested in all 32 GSC cultures (n=292) were included. Some GSC cultures were excluded due to insufficient material or poor cell growth. B) Robustness of GSC clustering into highly drug-sensitive and highly drug-resistant groups using the pvclust package in R. Multi-scale bootstrap resampling (n=10,000) was used to calculate p-values (%) for cluster stability, quantifying the reliability of the selected cultures per cluster. Approximately unbiased (AU) *p*-values (red) indicate cluster robustness across all resamplings, with AU > 50% considered reliable and AU ≥ 95% as highly significant. Bootstrap probability (BP) *p*-values are shown in green.

Additional file 4. *Drug sensitivity scores.*Complete data set of the drug sensitivity scores used for clustering the GSC cultures into highly drug-sensitive and highly drug-resistant groups.

Additional file 5. *Comparing proliferation using EdU incorporation between drug-resistant and drug-sensitive GSCs.* A-B) Overlaid histograms of the drug-resistant and drug-sensitive GSC cultures. The fluorescence signal (EdU-Alexa488) is plotted against cell count, showing the background fluorescence (left peak) and the EdU-positive cell fraction (right peak) with their respective percentages (%). A table summarizing the single cell fraction (%) and the EdU-positive cell fraction (%) in each culture is shown. C) The average fraction of EdU-positive cells between the two groups. Individual points represent each GSC culture.

Additional file 6. *Subtype classification of GSC cultures by ssGSEA and PCA analysis*. A) Classification of GSC cultures into proneural (PN) and mesenchymal (MES) subtypes using single-sample gene set enrichment analysis (ssGSEA) with a GSC-specific PN/MES gene set (n=55). ssGSEA enrichment scores calculated from PN and MES gene signatures are shown for each sample (left). Principal component analysis (PCA) based on the same gene set (right) demonstrates subtype-specific clustering along PC1 and PC2, with 95% confidence ellipses and points colored according to ssGSEA-derived classifications. B) The analysis repeated using a TCGA tissue-derived PN/MES gene set (n=352). ssGSEA enrichment scores for PN and MES signatures (left) and PCA visualization (right), demonstrate consistent subtype clustering, validating the robustness of classifications across independent gene sets. C) Score profiles of ssGSEA enrichment values for PN and MES gene signatures between drug-resistant and drug-sensitive GSC cultures.

Additional file 7. *Full-length Western blots*. Complete full-length Western blots used in the protein expression analysis of stemness markers, mature neural markers, and ECM-associated proteins.

Additional file 8. *Mutation profiles of drug-resistant and drug-sensitive GSC cultures*. Somatic mutations identified by whole-exome sequencing, filtered for Tier I-III genes with an allele frequency > 5%. Mutations are classified by type, including missense, nonsense, splice region single-nucleotide variants (SNV), splice region deletions, and frameshift mutations, in each GSC culture. Samples with two distinct mutations are split diagonally, with each half representing a different mutation type.

Additional file 9. *Annotation of somatic variants*. Summary of detected mutations in all GSC cultures, including the affected gene, predicted consequence at the transcript and protein level, variant class, clinical tier, and the corresponding genomic change (GRCh38 coordinates). All variants identified are classified as Tier III, indicating variants of uncertain clinical significance.

Additional file 10. *Differentially expressed genes*. Complete list of the differentially expressed genes between the drug-resistant and drug-sensitive GSC cultures.

Additional file 11. *Gene set enrichment analysis (GSEA) of differentially expressed genes.* A) Gene sets most significantly enriched among the differentially expressed genes between drug-resistant and drug-sensitive GSCs. BP = biological process, CC = cellular component, MF = molecular function. Significant terms are consistent with gene ontology enrichment analysis, showing collagen trimer (padj=2.7x10-13) and extracellular matrix (ECM) structural constituent (padj=4.6x10-9) with the highest associated gene counts . B) Network of the most significantly enriched pathways, including collagen-containing ECM (padj=2.7x10-13), external side of plasma membrane (padj=2.5x10-12), positive regulation of angiogenesis (padj=9.8x10-11), positive regulation of vasculature development (padj=9.8x10-11), and epithelium migration (padj=1.6x10-9). Nodes represent genes, with fold change values showing upregulation in drug-resistant GSCs.

Additional file 12. *Individual expression levels of the identified ECM and COL-associated genes in drug-resistant and drug-sensitive GSC cultures*. Individual expression levels of the A) genes (n=34) within the most enriched extracellular matrix (ECM) and collagen (COL) gene ontology terms, and B) differentially expressed COL genes (n=15) between drug-resistant and drug-sensitive GSC cultures. Gene expression levels are shown in the y-axis, with singular points as individual GSC cultures.

Additional file 13. *MGMT promoter and global CpG island methylation profiles in drug-resistant and drug-sensitive GSC cultures*. A) Methylation profile of the GSC cultures across all 98 CpG sites in the MGMT promoter. The CpG sites 76-79 are highlighted, which are traditionally used in clinical MGMT pyrosequencing. From top to bottom, the plot shows the full MGMT CpG island (chr10: 129466685-129467446, hg38), modified bases (5mC) as closed black circles, raw log-likelihood ratios (0-1), and the smoothened fraction plot with methylation percentages (0 (0%) - 1 (100%)). GSC cultures are grouped by color. B) Global methylation level, averaged from all the CpG islands (n=27941) between drug-resistant and drug-sensitive GSC cultures. Individual points represent each GSC culture. C) Methylation levels of each differentially methylated CpG island (n=172), alongside their associated genes. Each point represents the mean methylation in each group, with error bars as means with SD.

Additional file 14. *Methylation levels of CpG islands linked to genes involved in axonogenesis and cell leading edge dynamics and their corresponding gene expression levels*. A) Methylation levels of the CpG islands (n=24) linked to genes within the axonogenesis and cell leading edge ontology terms in drug-resistant and drug-sensitive GSC cultures. Individual points represent each GSC culture. B) Expression levels of the corresponding genes (*n*=20) in both groups. Error bars represent means with SD.

## Data Availability

Data supporting the findings of this study, including drug sensitivity scores, differential expression, and individual gene expression and methylation levels, are included in this published article and its additional files. All non-sensitive data, including global RNA-seq counts, DNA methylation matrix across CpG islands and full per-coordinate CpG site methylation data for all individual samples, have also been made publicly available in a GitHub repository (https:/github.com/EstabilloLL/GBM_IntrinsicDrugResistance). Other data and code used in the current study are available from the corresponding author upon reasonable request.

## Abbreviations

GBM: Glioblastoma.
TMZ: Temozolomide.
GSC: Glioblastoma stem cell.
ABC: ATP-binding cassette.
TME: Tumor microenvironment.
DSS: Drug sensitivity score.
EdU: 5-ethynyl-2’-deoxyuridine.
DGE: Differential gene expression.
PCA: Principal component analysis.
ORA: Over-representation analysis.
GO: Gene ontology.
COL: Collagen.
ECM: Extracellular matrix.
GSEA: Gene set enrichment analysis.
5mC: 5-methylcytosine.
