# Supplementary figures and images for: Drug resistant glioblastoma stem cells exhibit enriched stemness signatures and share extracellular matrix overexpression
Source: BMC Cancer. 2025 Oct 27;25:1655. doi: 10.1186/s12885-025-15163-z (PMC12560458; doi:10.1186/s12885-025-15163-z)

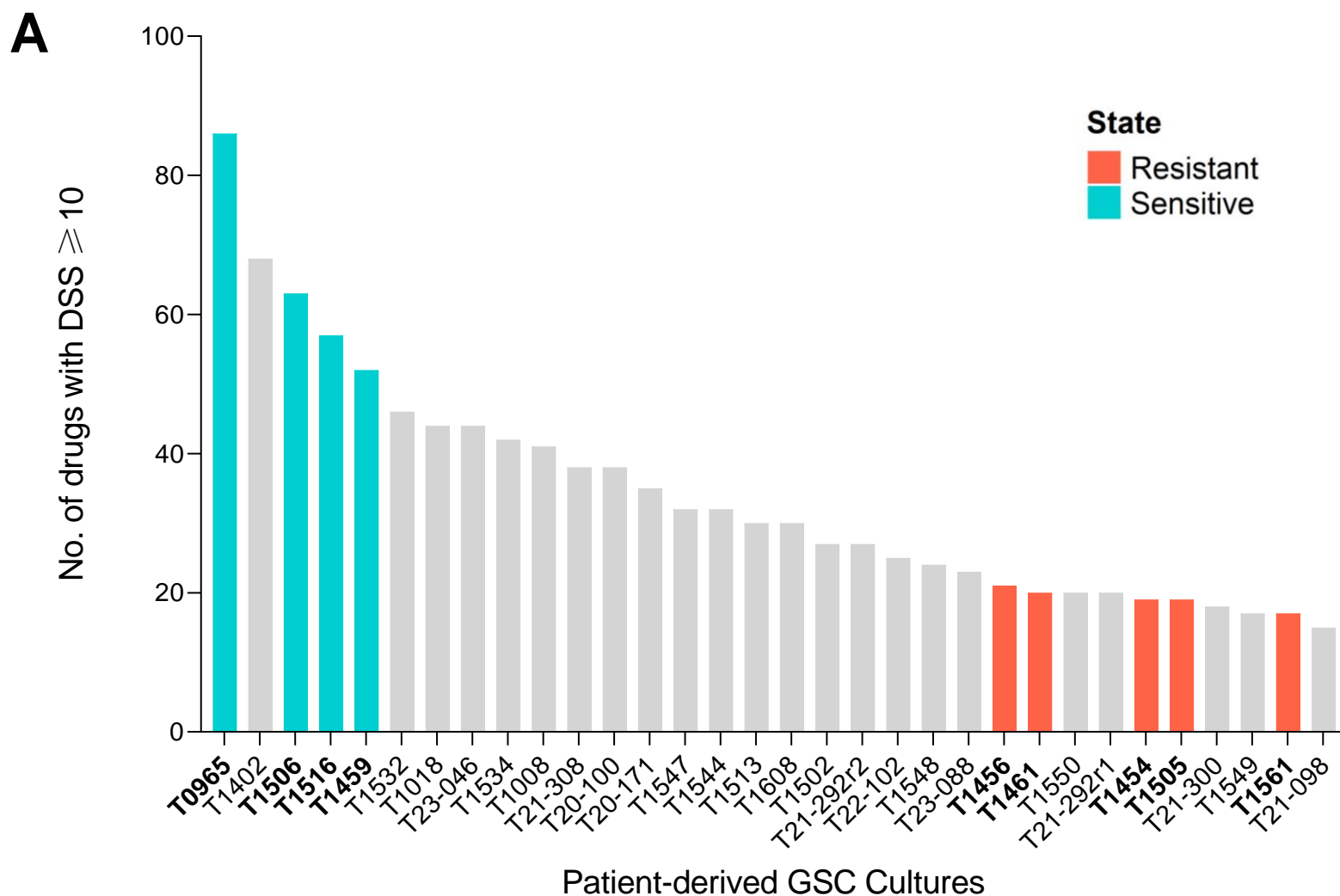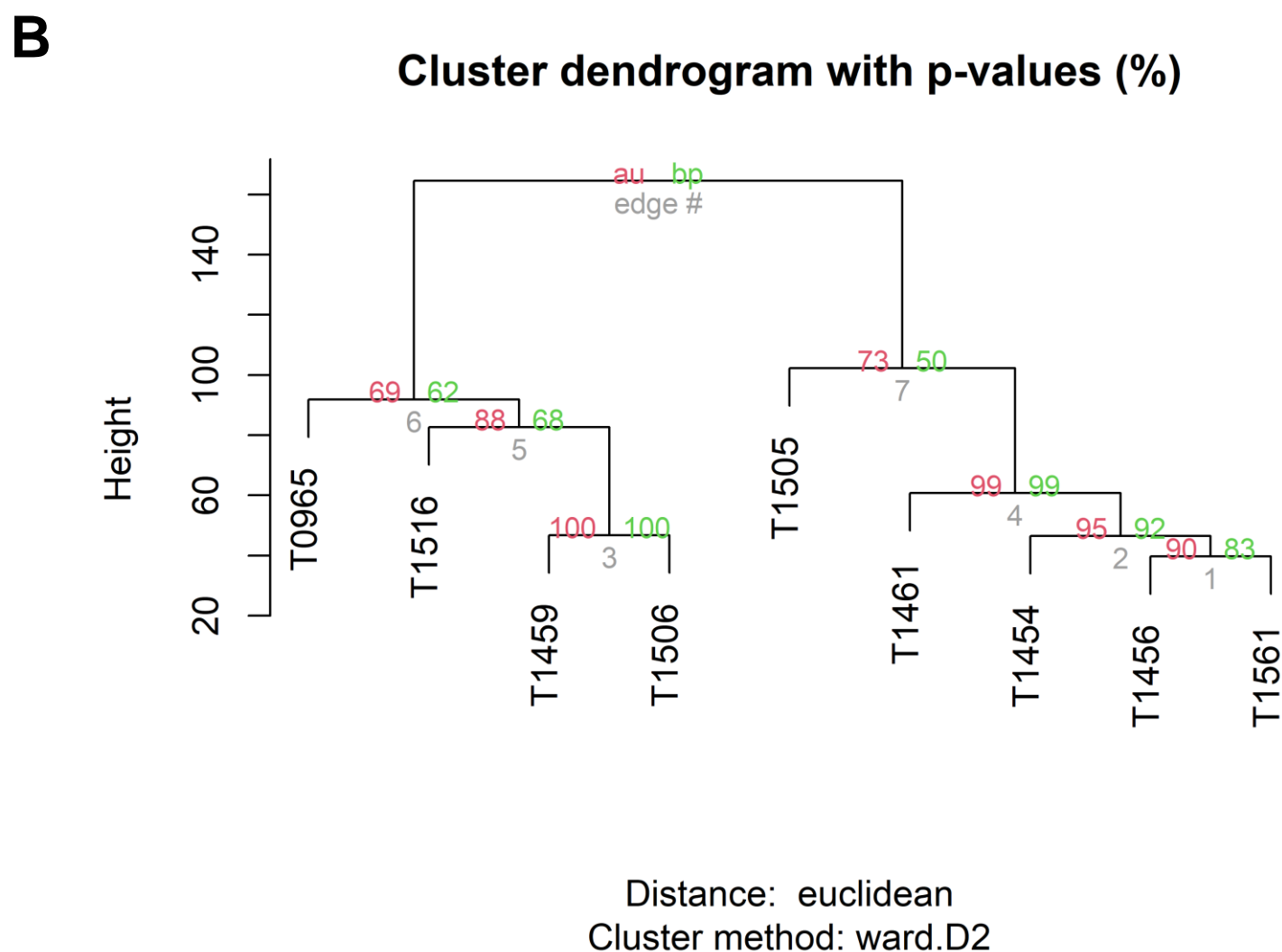

Supplement: Supplementary file 3 — Additional file 3. Clustering of GSCs according to drug sensitivity. A) Ranking from most drug-sensitive to most drug-resistant of 32 patient-derived GSC cultures screened against ~up to 527 anti-cancer drugs. The GSCs are ranked based on the number of drugs with DSS ≥ 10. Only drugs tested in all 32 GSC cultures (n=292) were included. Some GSC cultures were excluded due to insufficient material or poor cell growth. B) Robustness of GSC clustering into highly drug-sensitive and highly drug-resistant groups using the pvclust package in R. Multi-scale bootstrap resampling (n=10,000) was used to calculate p-values (%) for cluster stability, quantifying the reliability of the selected cultures per cluster. Approximately unbiased (AU) p-values (red) indicate cluster robustness across all resamplings, with AU > 50% considered reliable and AU ≥ 95% as highly significant. Bootstrap probability (BP) p-values are shown in green. [file 12885_2025_15163_MOESM3_ESM.pdf]

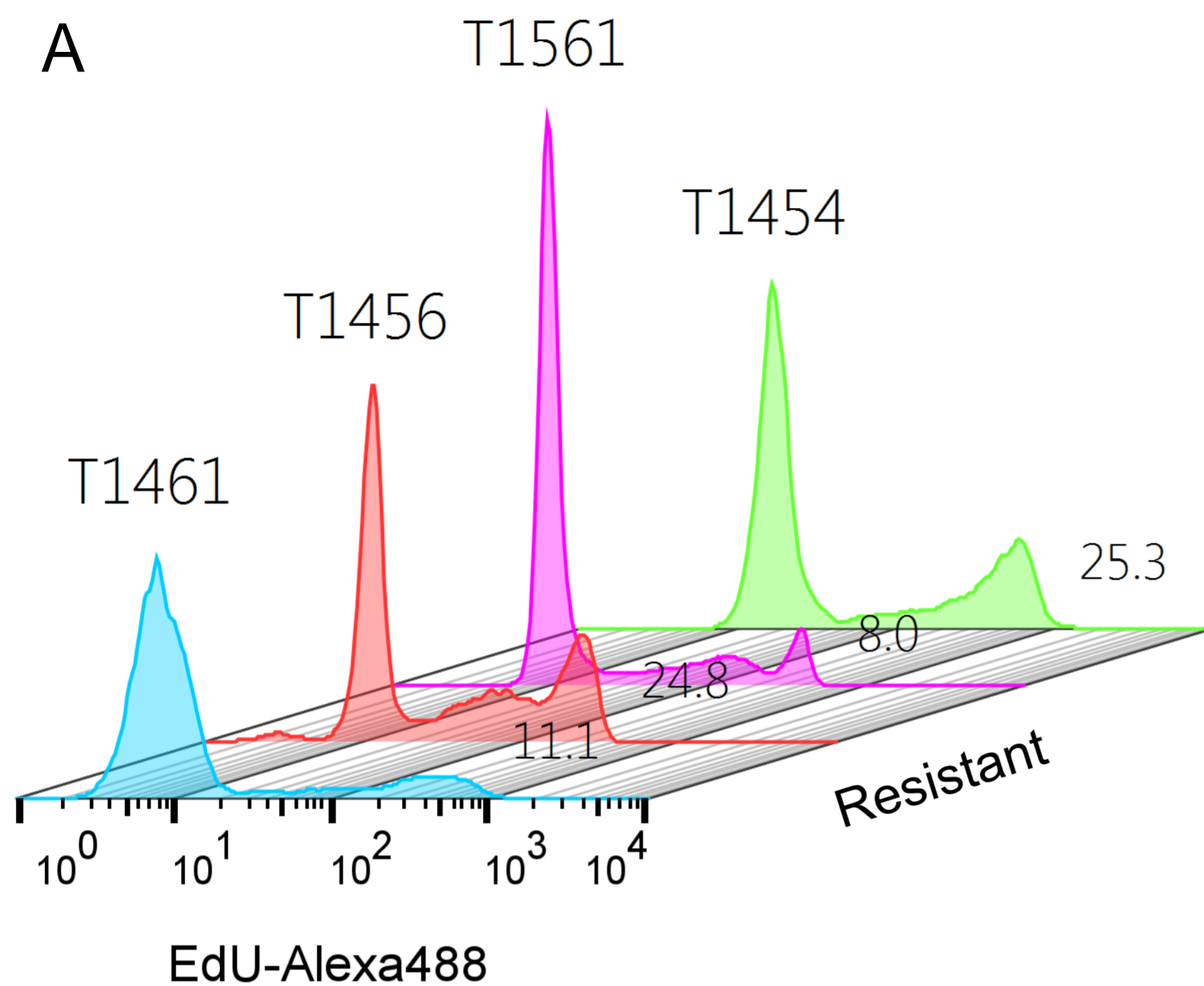

|  | ID    | Single Cell Fraction (%) | EdU-positive Fraction (%) |
|--|-------|--------------------------|---------------------------|
|  | T1461 | 83.0                     | 11.1                      |
|  | T1456 | 99.5                     | 24.8                      |
|  | T1561 | 86.8                     | 8.0                       |
|  | T1454 | 99.5                     | 25.3                      |
|  | T1516 | 98.5                     | 13.3                      |
|  | T1506 | 97.9                     | 4.7                       |
|  | T1459 | 99.1                     | 14.3                      |
|  | T0965 | 97.3                     | 15.3                      |

**State**

Resistant

Sensitive

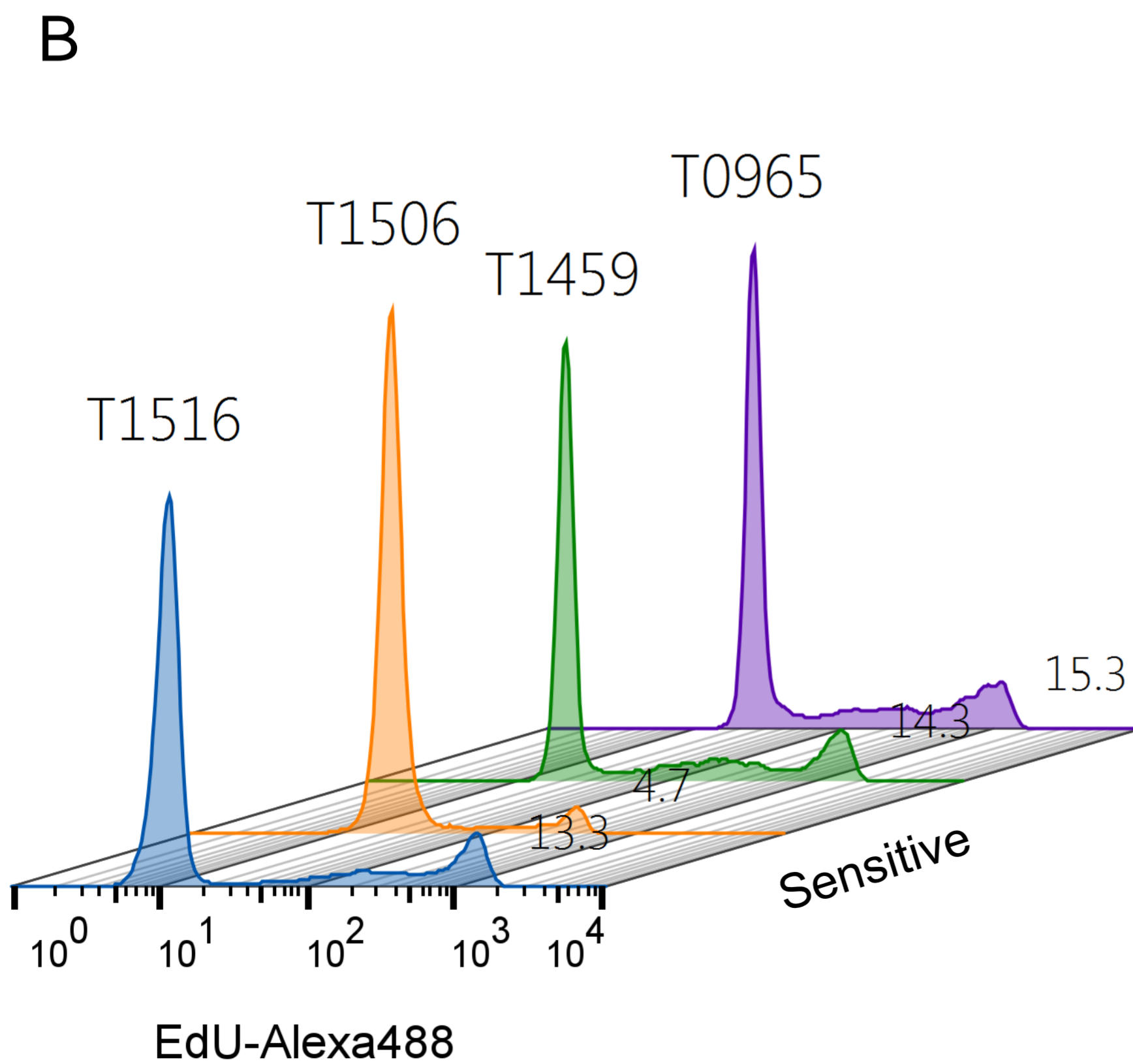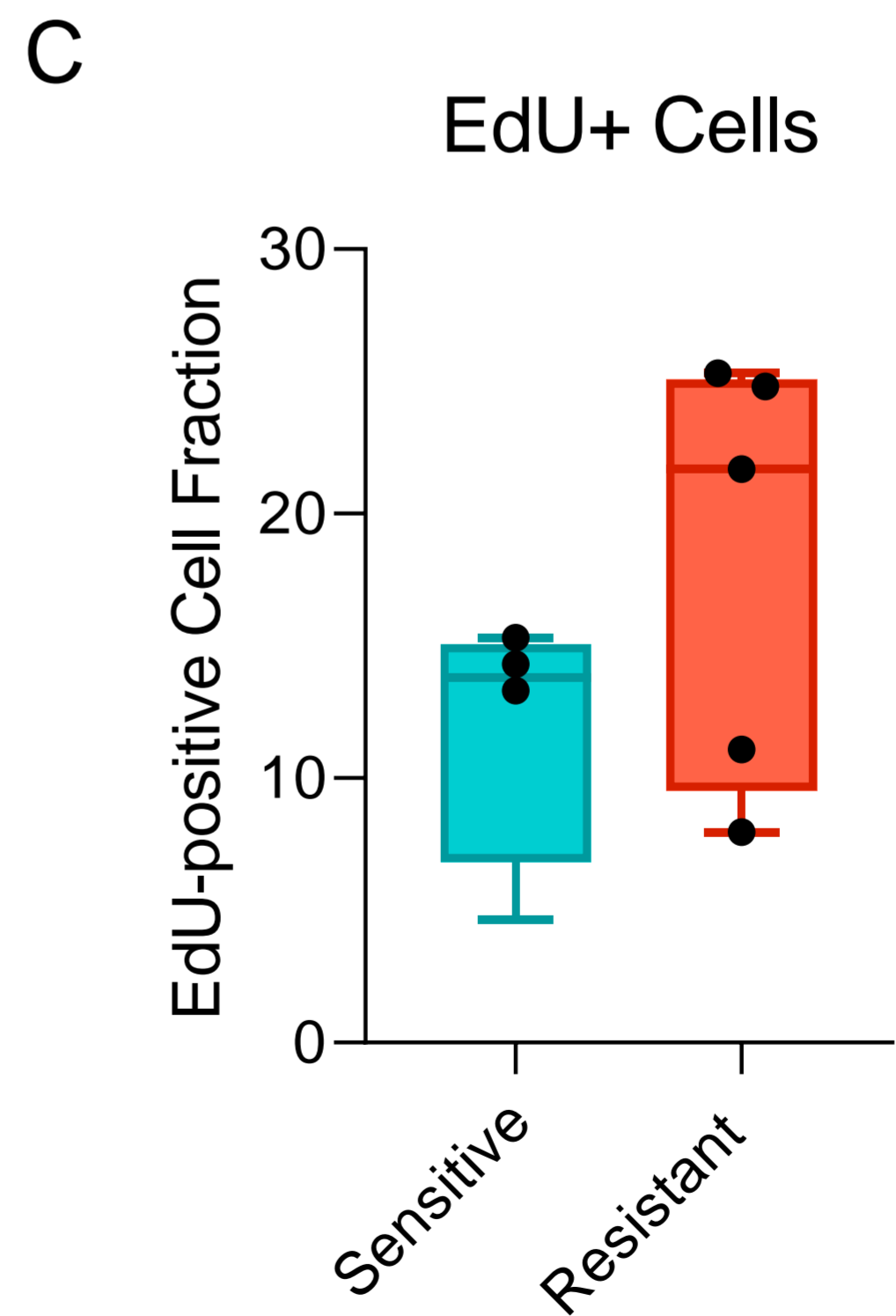

Supplement: Supplementary file 5 — Additional file 5. Comparing proliferation using EdU incorporation between drug-resistant and drug-sensitive GSCs. A-B) Overlaid histograms of the drug-resistant and drug-sensitive GSC cultures. The fluorescence signal (EdU-Alexa488) is plotted against cell count, showing the background fluorescence (left peak) and the EdU-positive cell fraction (right peak) with their respective percentages (%). A table summarizing the single cell fraction (%) and the EdU-positive cell fraction (%) in each culture is shown. C) The average fraction of EdU-positive cells between the two groups. Individual points represent each GSC culture. [file 12885_2025_15163_MOESM5_ESM.pdf]

A

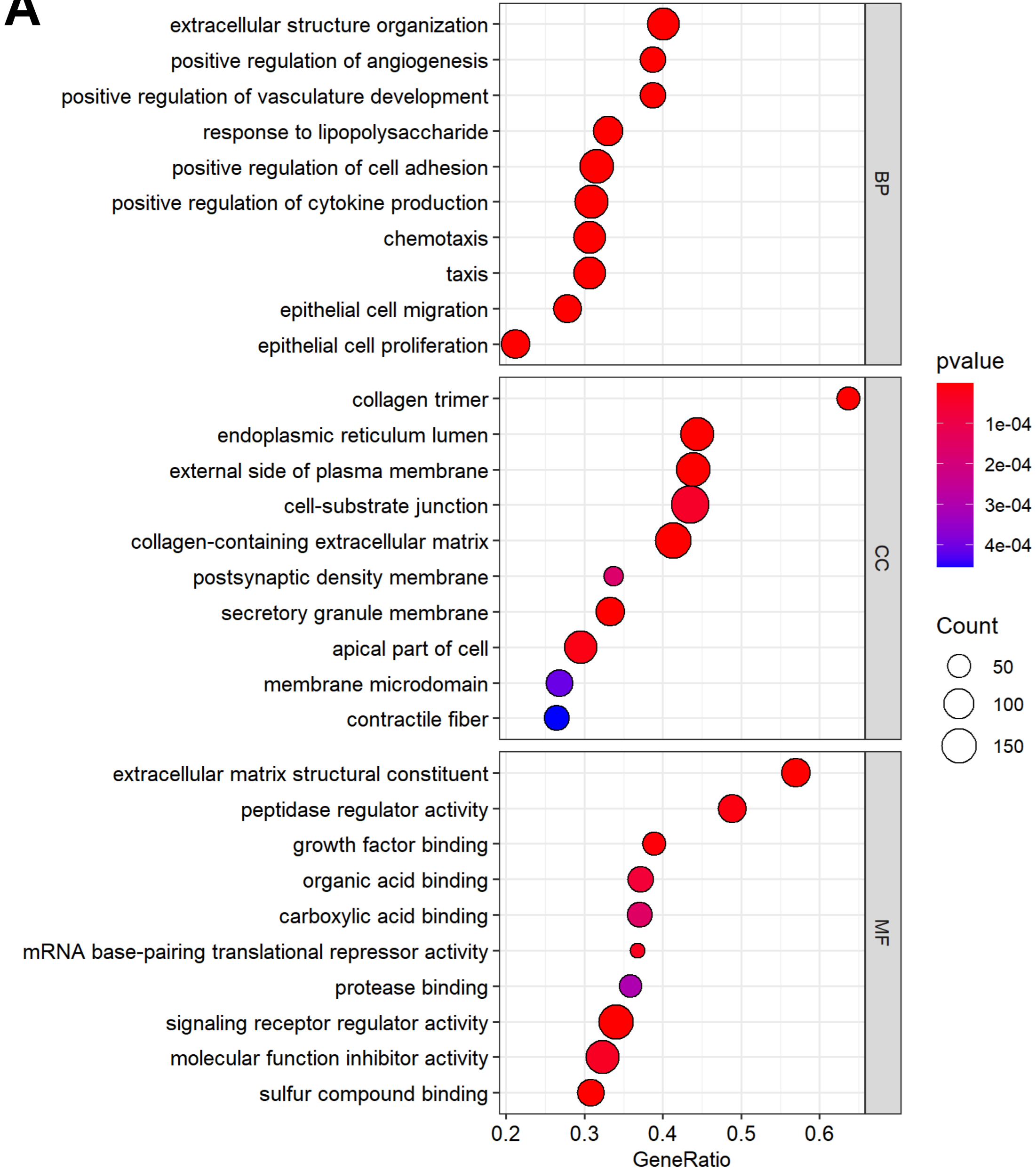

B

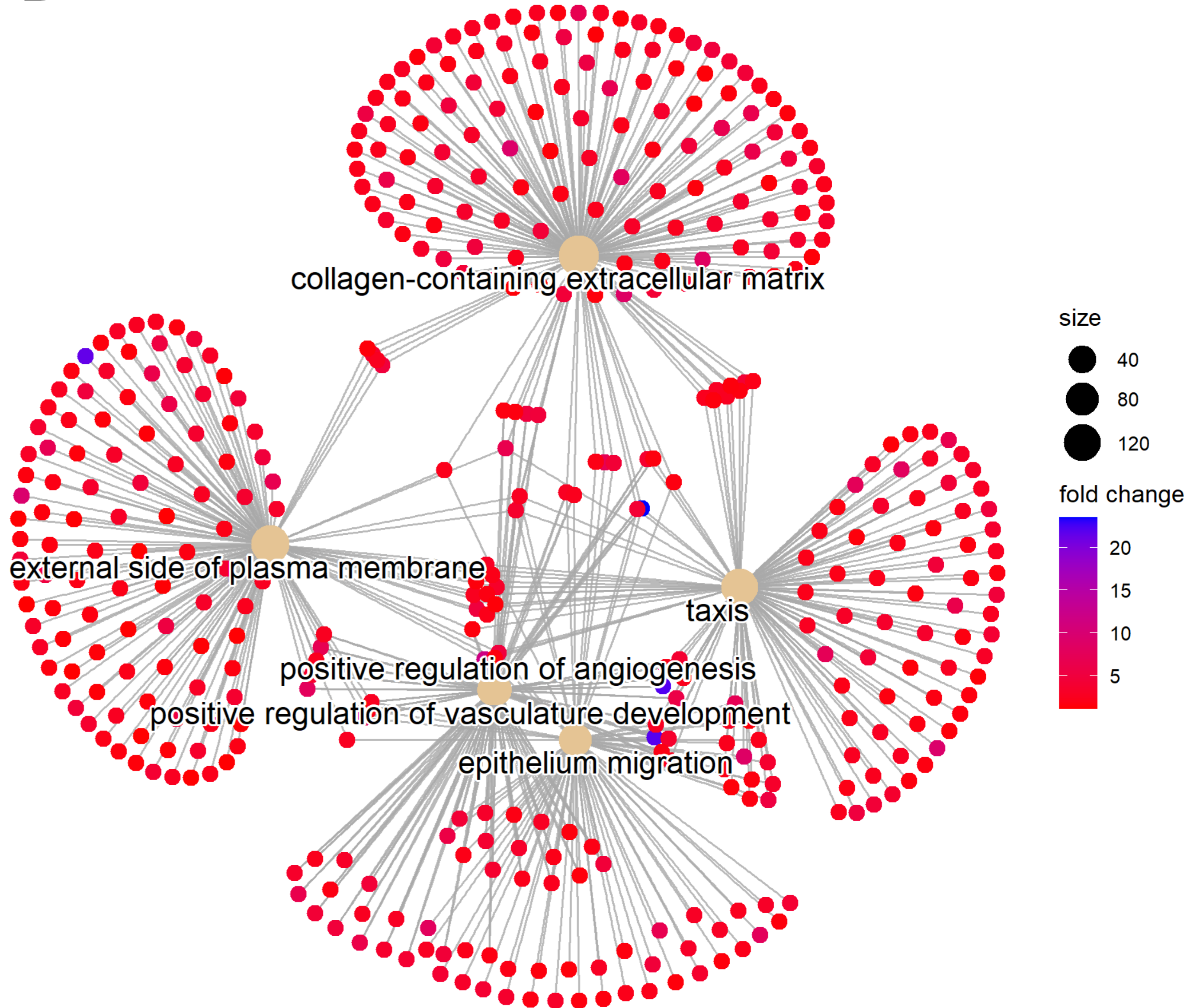

Supplement: Supplementary file 11 — Additional file 11. Gene set enrichment analysis (GSEA) of differentially expressed genes. A) Gene sets most significantly enriched among the differentially expressed genes between drug-resistant and drug-sensitive GSCs. BP = biological process, CC = cellular component, MF = molecular function. Significant terms are consistent with gene ontology enrichment analysis, showing collagen trimer (padj=2.7x10^-13^) and extracellular matrix (ECM) structural constituent (padj=4.6x10^-9^) with the highest associated gene counts . B) Network of the most significantly enriched pathways, including collagen-containing ECM (padj=2.7x10^-13^), external side of plasma membrane (padj=2.5x10^-12^), positive regulation of angiogenesis (padj=9.8x10^-11^), positive regulation of vasculature development (padj=9.8x10^-11^), and epithelium migration (padj=1.6x10^-9^). Nodes represent genes, with fold change values showing upregulation in drug-resistant GSCs. [file 12885_2025_15163_MOESM11_ESM.pdf]

**A**

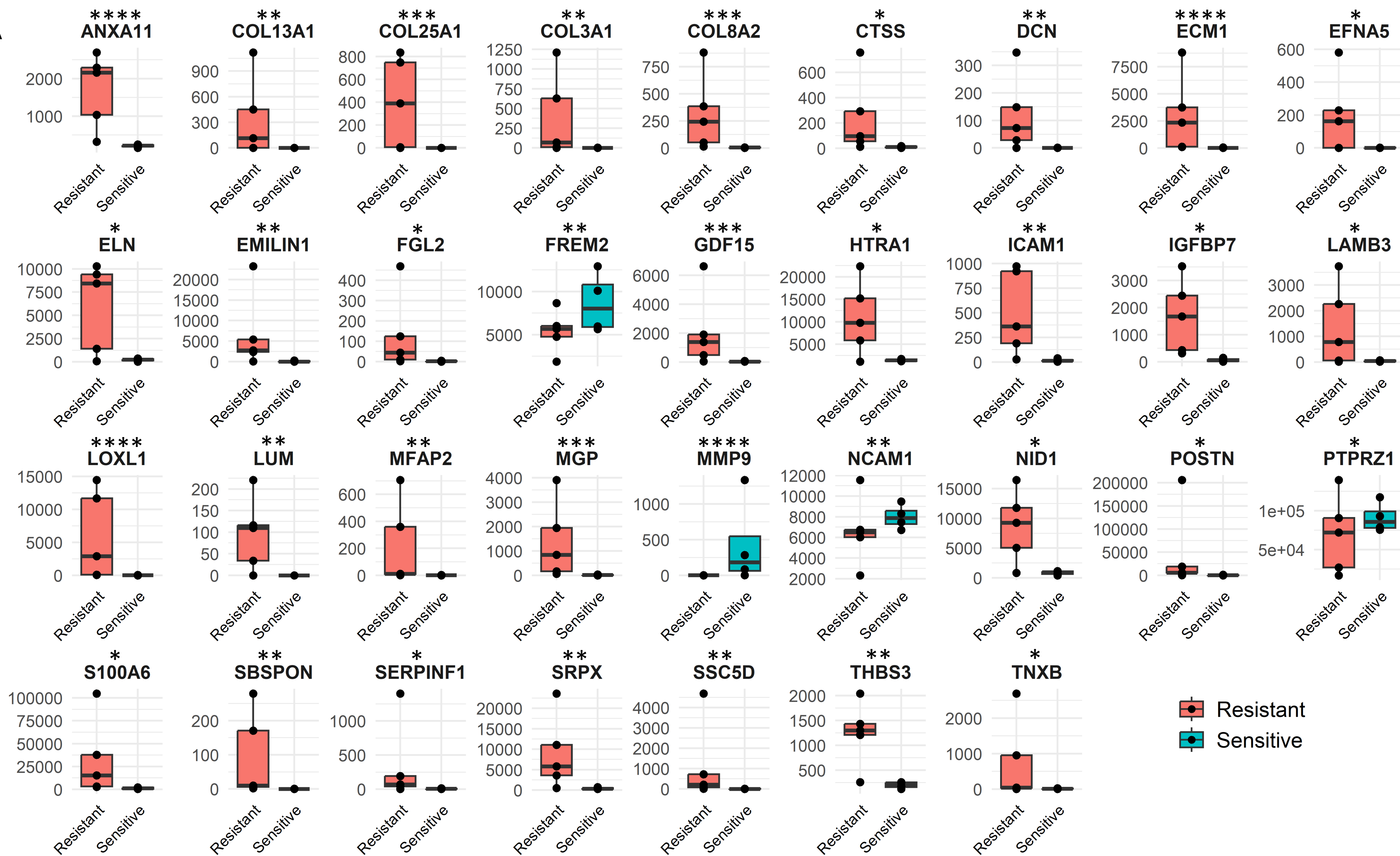

**B**

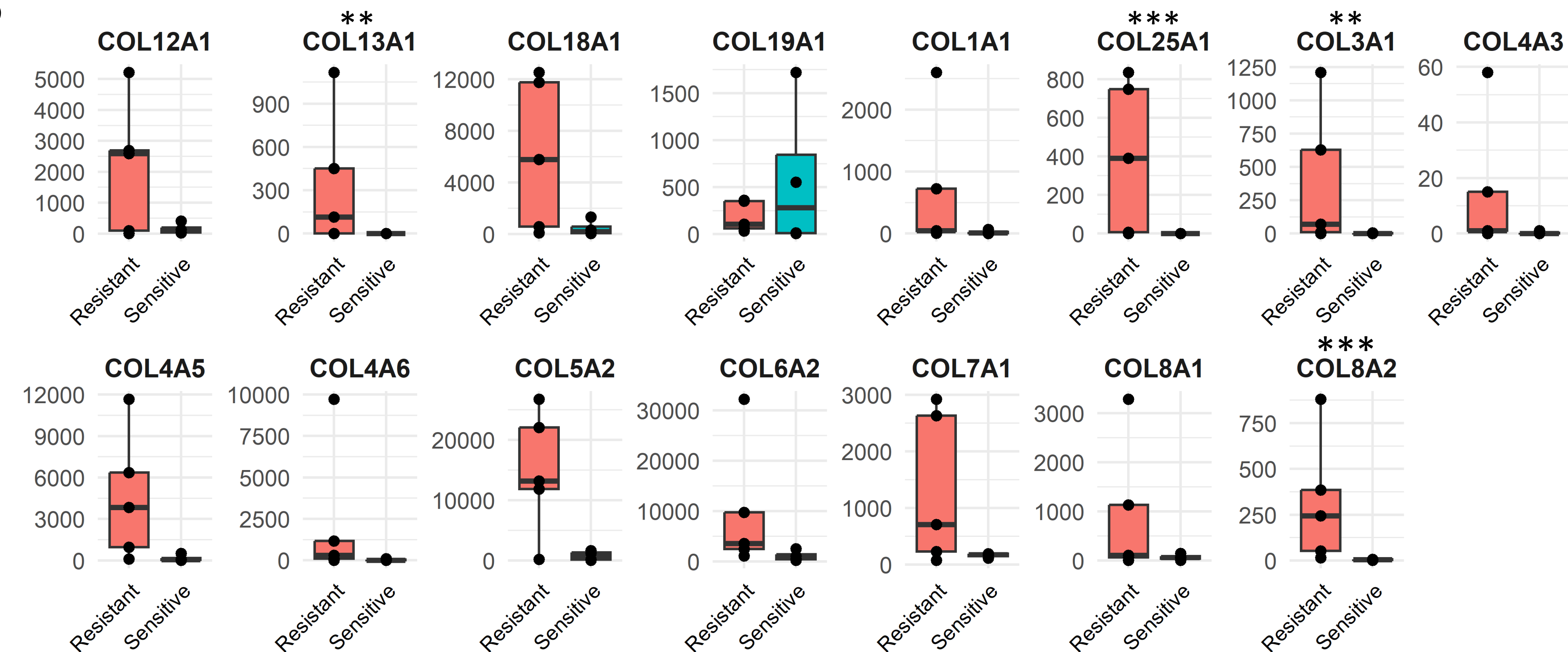

Supplement: Supplementary file 12 — Additional file 12. Individual expression levels of the identified ECM and COL-associated genes in drug-resistant and drug-sensitive GSC cultures. Individual expression levels of the A) genes (n=34) within the most enriched extracellular matrix (ECM) and collagen (COL) gene ontology terms, and B) differentially expressed COL genes (n=15) between drug-resistant and drug-sensitive GSC cultures. Gene expression levels are shown in the y-axis, with singular points as individual GSC cultures. [file 12885_2025_15163_MOESM12_ESM.pdf]

A

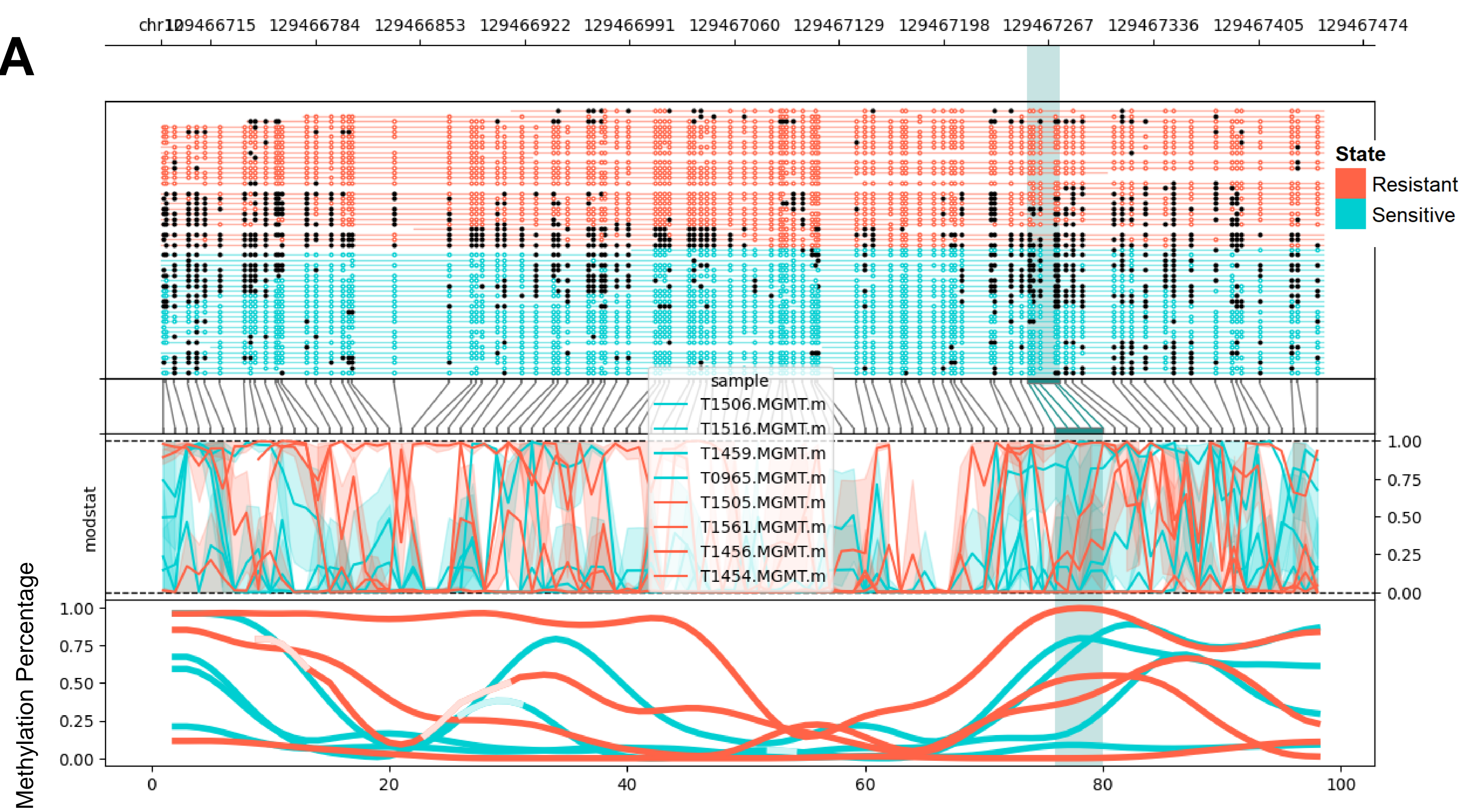

B

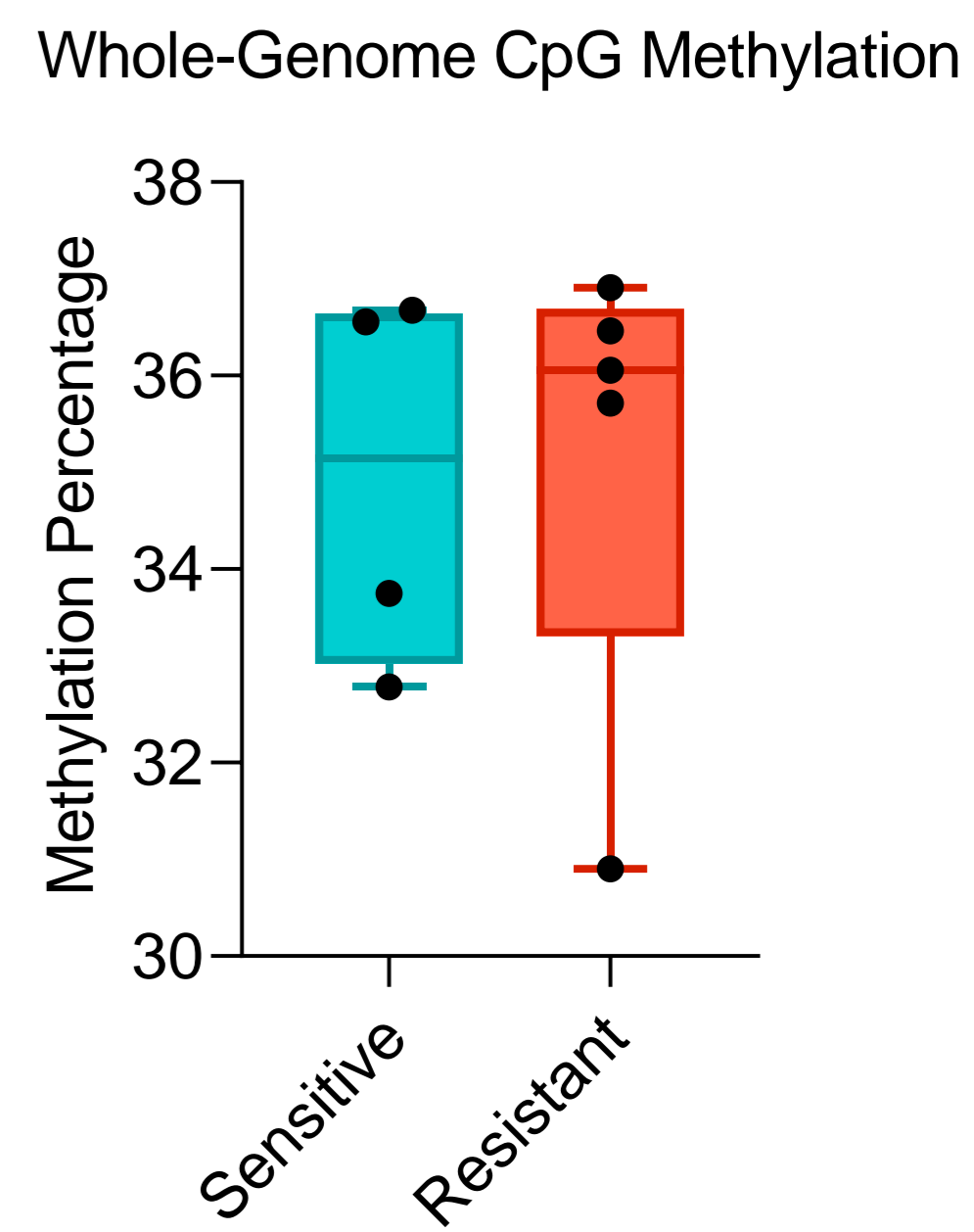

C

### Methylation Percentage by CpG Island

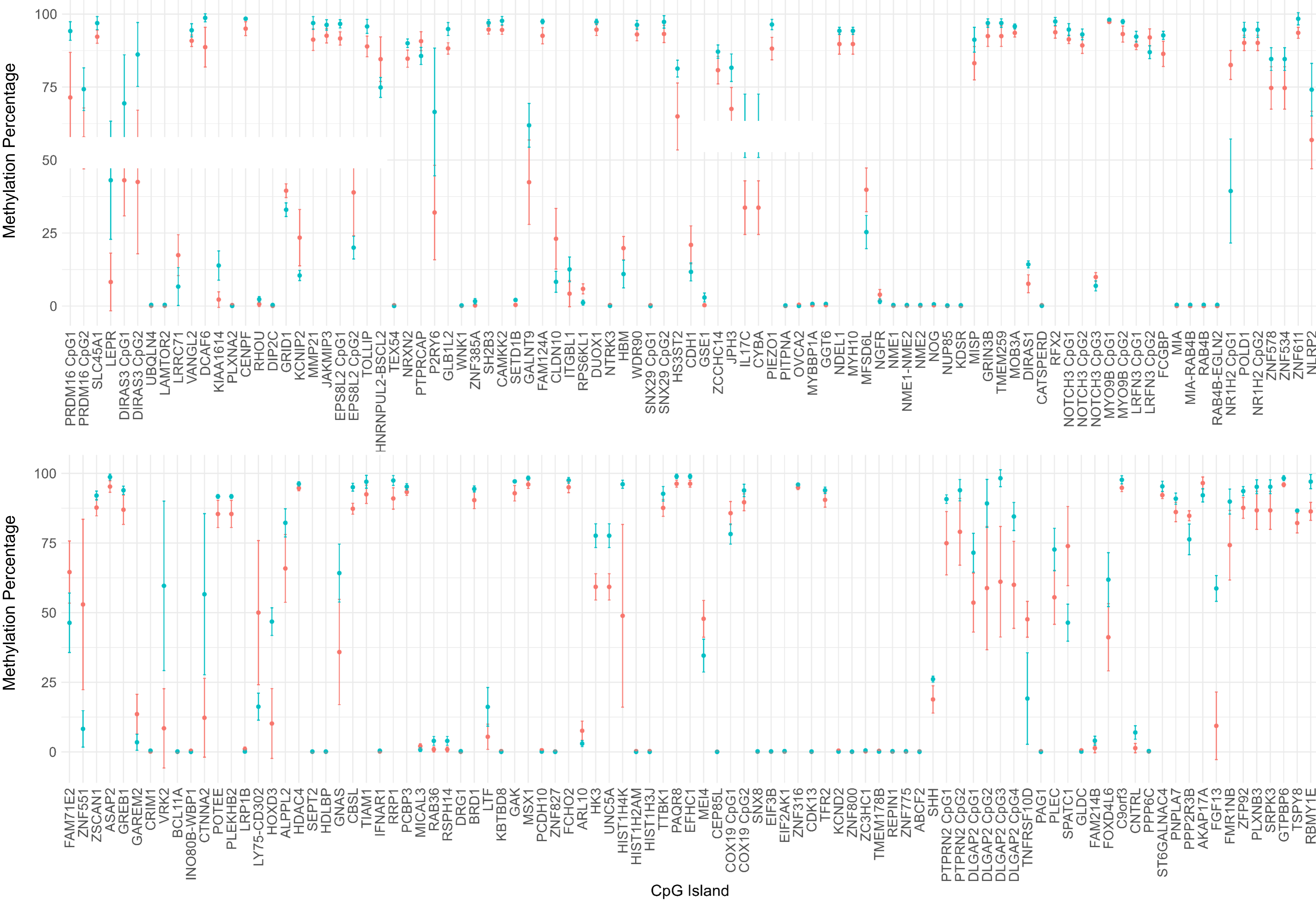

Supplement: Supplementary file 13 — Additional file 13. MGMT promoter and global CpG island methylation profiles in drug-resistant and drug-sensitive GSC cultures. A) Methylation profile of the GSC cultures across all 98 CpG sites in the MGMT promoter. The CpG sites 76-79 are highlighted, which are traditionally used in clinical MGMT pyrosequencing. From top to bottom, the plot shows the full MGMT CpG island (chr10: 129466685-129467446, hg38), modified bases (5mC) as closed black circles, raw log-likelihood ratios (0-1), and the smoothened fraction plot with methylation percentages (0 (0%) - 1 (100%)). GSC cultures are grouped by color. B) Global methylation level, averaged from all the CpG islands (n=27941) between drug-resistant and drug-sensitive GSC cultures. Individual points represent each GSC culture. C) Methylation levels of each differentially methylated CpG island (n=172), alongside their associated genes. Each point represents the mean methylation in each group, with error bars as means with SD. [file 12885_2025_15163_MOESM13_ESM.pdf]

A

Methylation Percentage

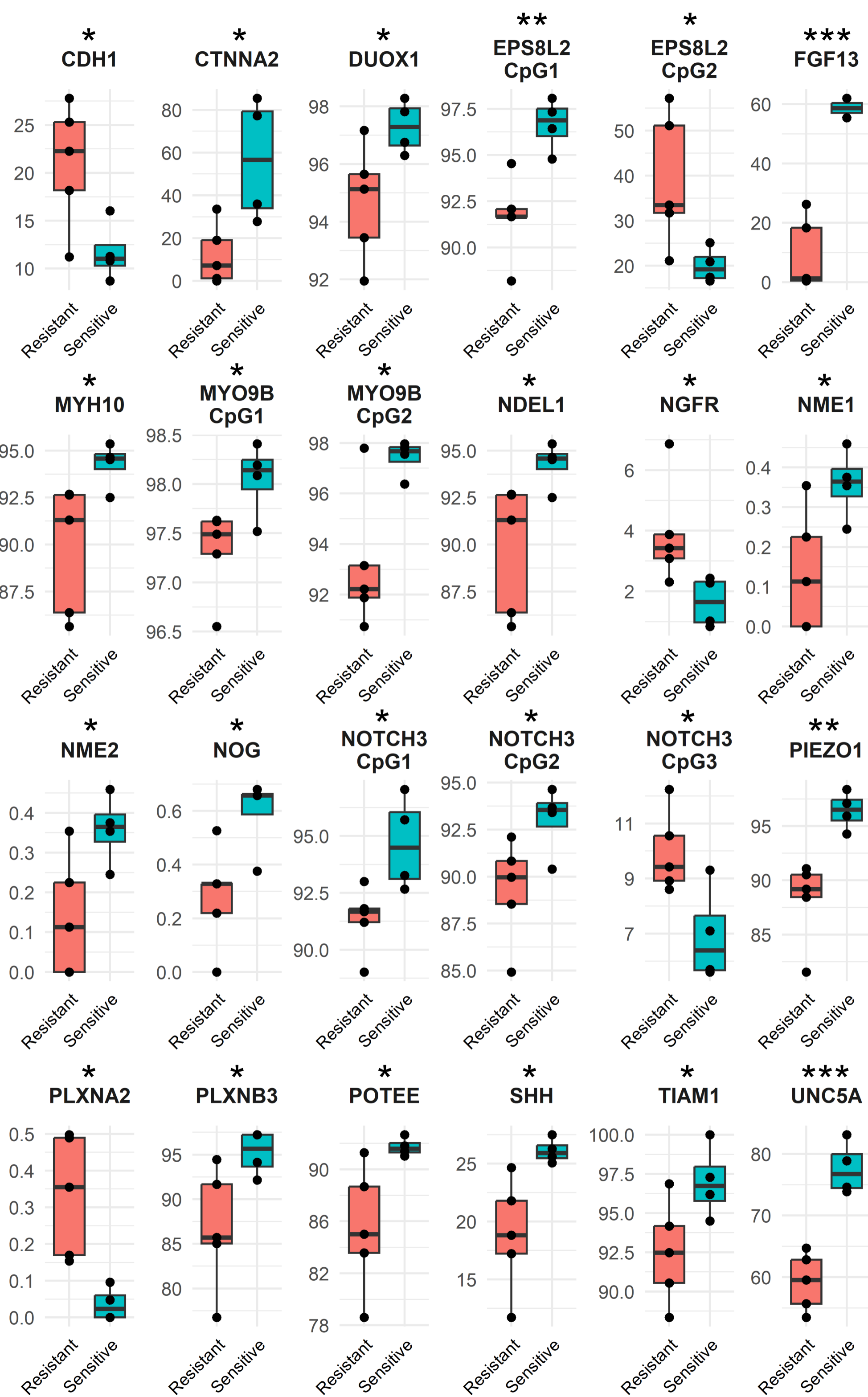

B

Gene Expression Level (Normalized Counts)

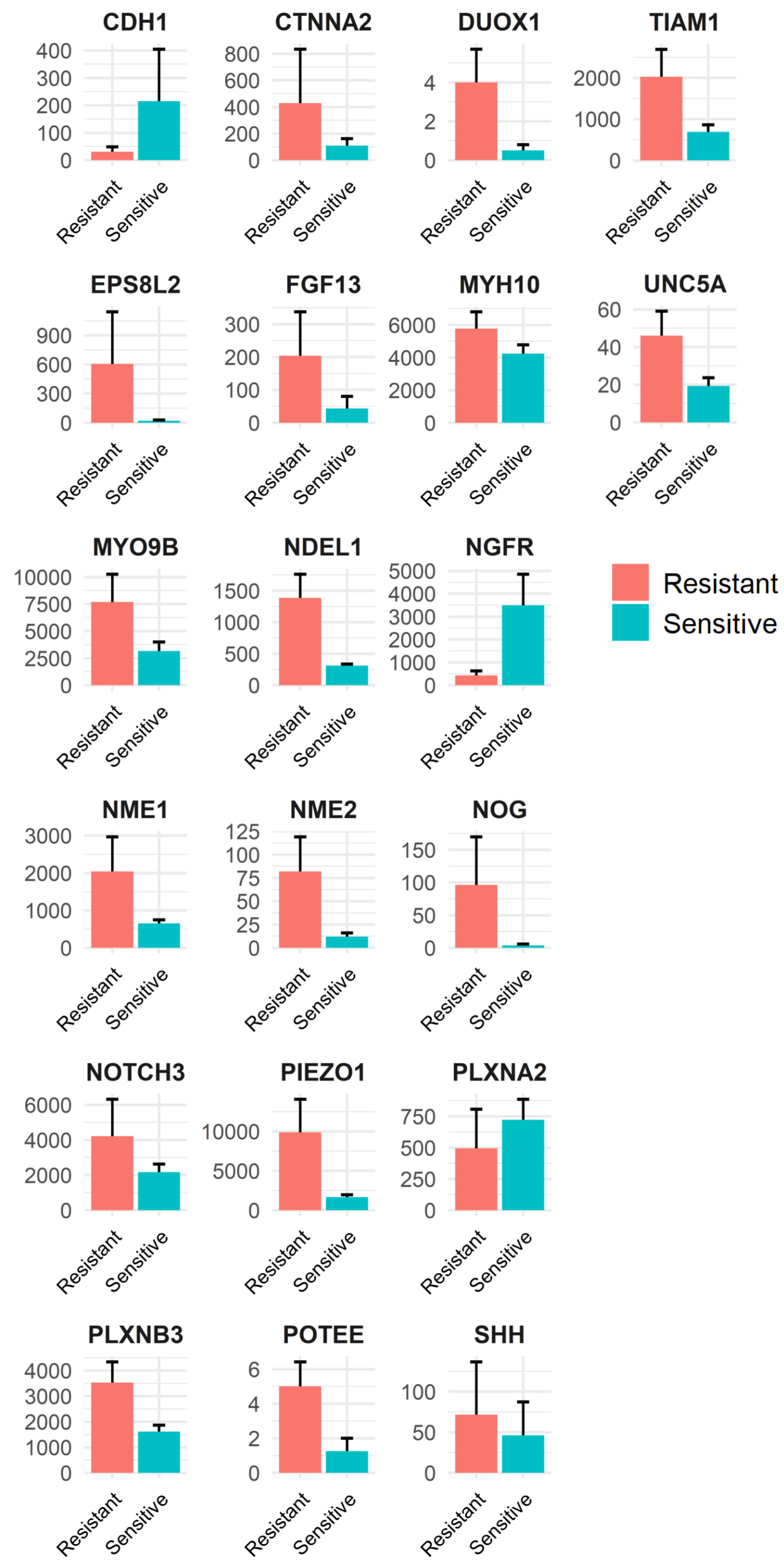

Supplement: Supplementary file 14 — Additional file 14. Methylation levels of CpG islands linked to genes involved in axonogenesis and cell leading edge dynamics and their corresponding gene expression levels. A) Methylation levels of the CpG islands (n=24) linked to genes within the axonogenesis and cell leading edge ontology terms in drug-resistant and drug-sensitive GSC cultures. Individual points represent each GSC culture. B) Expression levels of the corresponding genes (n=20) in both groups. Error bars represent means with SD. [file 12885_2025_15163_MOESM14_ESM.pdf]
