# Supplementary material for: Drug resistant glioblastoma stem cells exhibit enriched stemness signatures and share extracellular matrix overexpression
Source: BMC Cancer. 2025 Oct 27;25:1655. doi: 10.1186/s12885-025-15163-z (PMC12560458; doi:10.1186/s12885-025-15163-z)

A

GBM Subtype Classification (ssGSEA)

Glioma Stem Cell-Adapted Gene Set

GBM	PN_Score	MES_Score	Subtype
T1516	0.50186276	-0.36176313	PN
T1459	0.56155812	-0.43844188	PN
T1506	0.49893294	-0.36874125	PN
T0965	0.4637864	-0.32416359	PN
T1461	0.39056723	-0.23277655	PN
T1561	0.4349498	-0.29074614	PN
T1456	0.33611204	-0.17620719	PN
T1454	0.01348201	0.16213242	MES
T1505	0.32248952	-0.16199313	PN

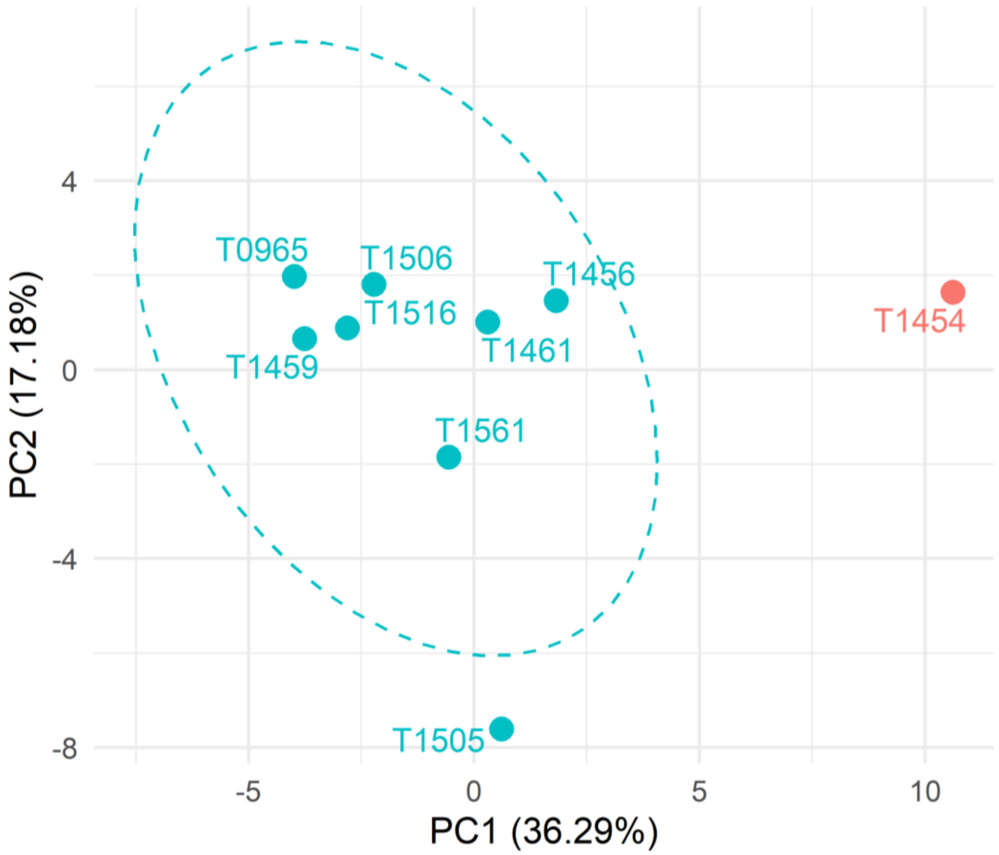

C

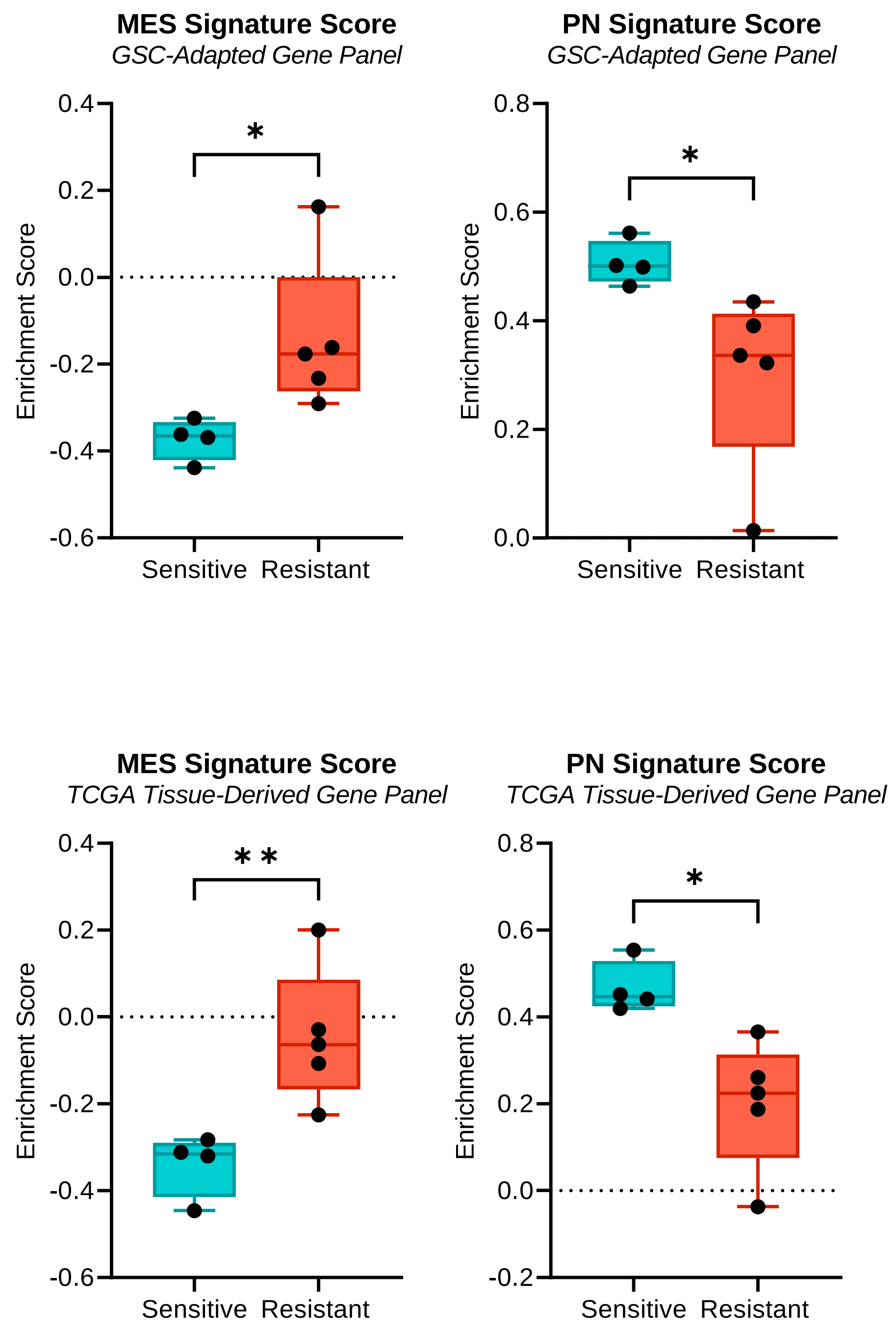

B

GBM Subtype Classification (ssGSEA)

TCGA Tissue-Derived Gene Set

GBM	PN_Score	MES_Score	Subtype
T1516	0,4516102	-0,31977375	PN
T1459	0,55430272	-0,44569728	PN
T1506	0,4193567	-0,28290044	PN
T0965	0,44146725	-0,31141256	PN
T1461	0,22478099	-0,06362033	PN
T1561	0,36546955	-0,22559478	PN
T1456	0,1876512	-0,02910044	PN
T1454	-0,03694853	0,20060315	MES
T1505	0,26105389	-0,10710885	PN

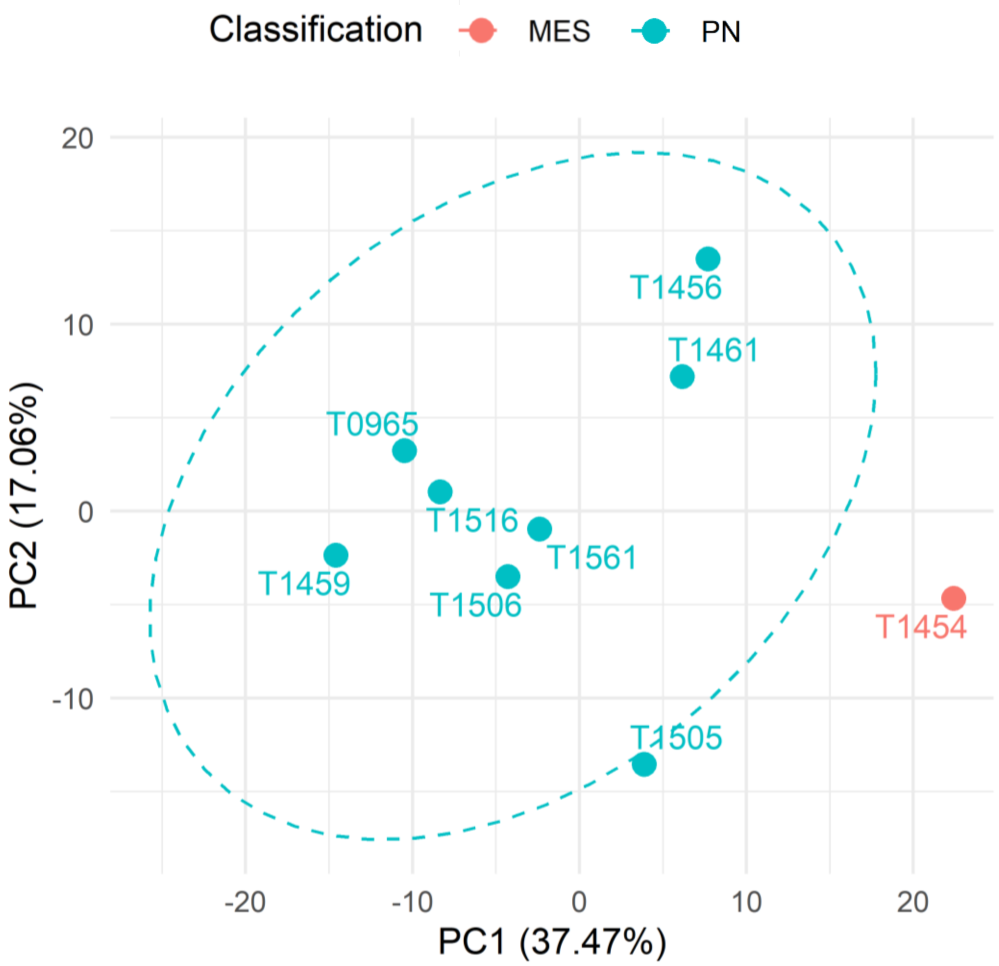

Supplement: Supplementary file 6 — Additional file 6. Subtype classification of GSC cultures by ssGSEA and PCA analysis. A) Classification of GSC cultures into proneural (PN) and mesenchymal (MES) subtypes using single-sample gene set enrichment analysis (ssGSEA) with a GSC-specific PN/MES gene set (n=55). ssGSEA enrichment scores calculated from PN and MES gene signatures are shown for each sample (left). Principal component analysis (PCA) based on the same gene set (right) demonstrates subtype-specific clustering along PC1 and PC2, with 95% confidence ellipses and points colored according to ssGSEA-derived classifications. B) The analysis repeated using a TCGA tissue-derived PN/MES gene set (n=352). ssGSEA enrichment scores for PN and MES signatures (left) and PCA visualization (right), demonstrate consistent subtype clustering, validating the robustness of classifications across independent gene sets. C) Score profiles of ssGSEA enrichment values for PN and MES gene signatures between drug-resistant and drug-sensitive GSC cultures. [file 12885_2025_15163_MOESM6_ESM.pdf]
