# Supplementary material for: Drug resistant glioblastoma stem cells exhibit enriched stemness signatures and share extracellular matrix overexpression
Source: BMC Cancer. 2025 Oct 27;25:1655. doi: 10.1186/s12885-025-15163-z (PMC12560458; doi:10.1186/s12885-025-15163-z)
Supplement: Supplementary file 7 — Additional file 7. Full-length Western blots. Complete full-length Western blots used in the protein expression analysis of stemness markers, mature neural markers, and ECM-associated proteins. [file 12885_2025_15163_MOESM7_ESM.pdf]

Fig 2B: OLIG2

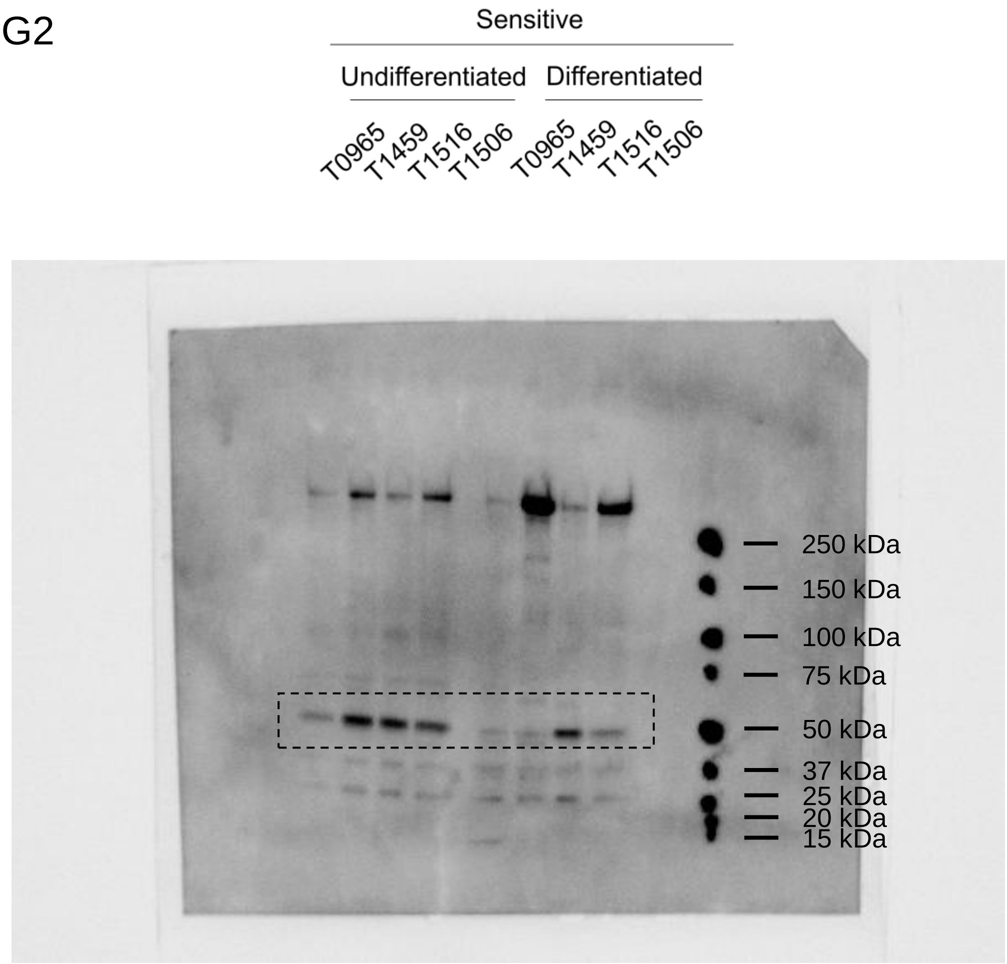

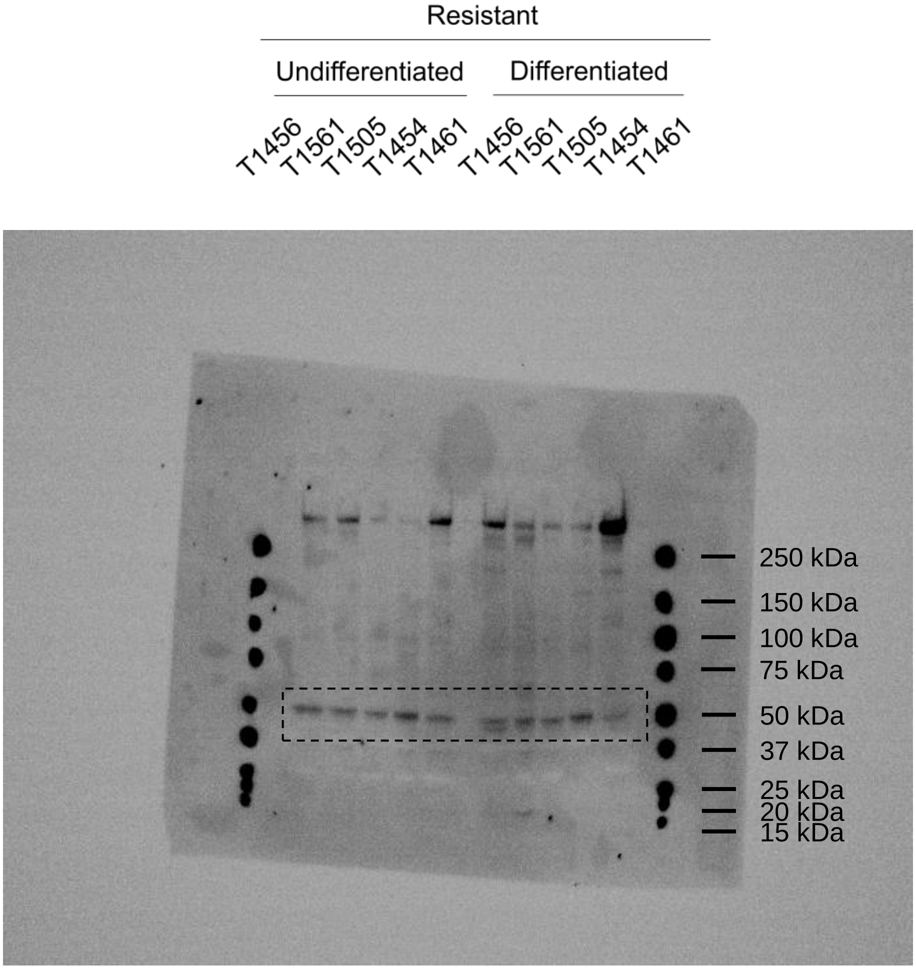

Supplementary Figure 1. *Full-length Western blot images for OLIG2 corresponding to Fig. 2B. The cropped region is indicated and the relevant molecular weights are labeled.*

Fig 2B: CD109

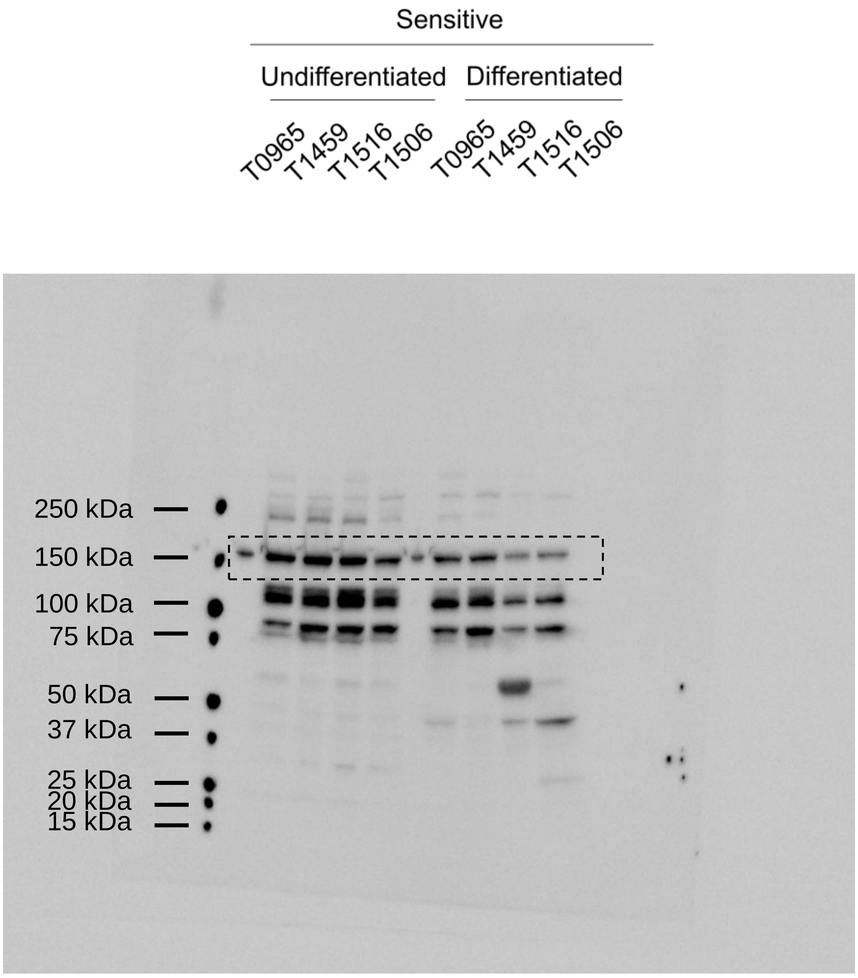

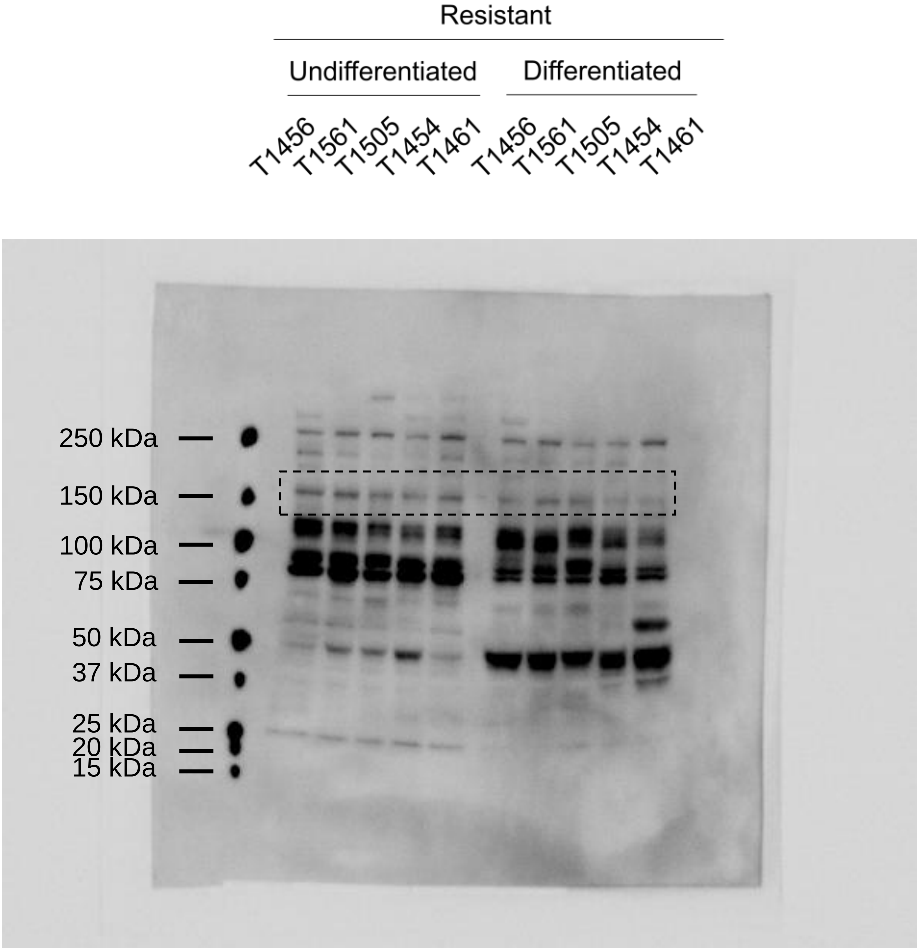

Supplementary Figure 2. *Full-length Western blot images for CD109 corresponding to Fig. 2B. The cropped region is indicated and the relevant molecular weights are labeled.*

Fig 2B: POU3F2

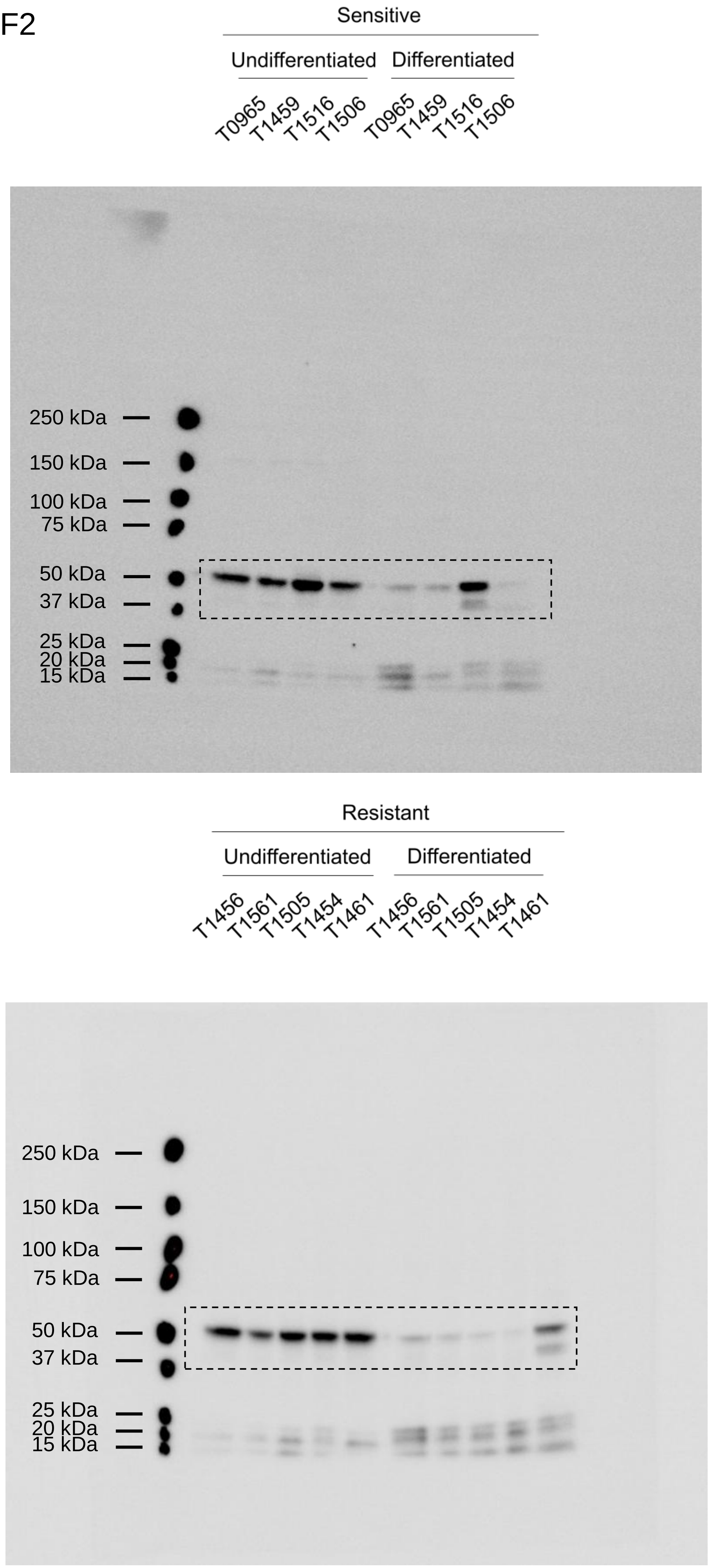

Supplementary Figure 3. *Full-length Western blot images for POU3F2 corresponding to Fig. 2B. The cropped region is indicated and the relevant molecular weights are labeled.*

Fig 2B: ITGβ1

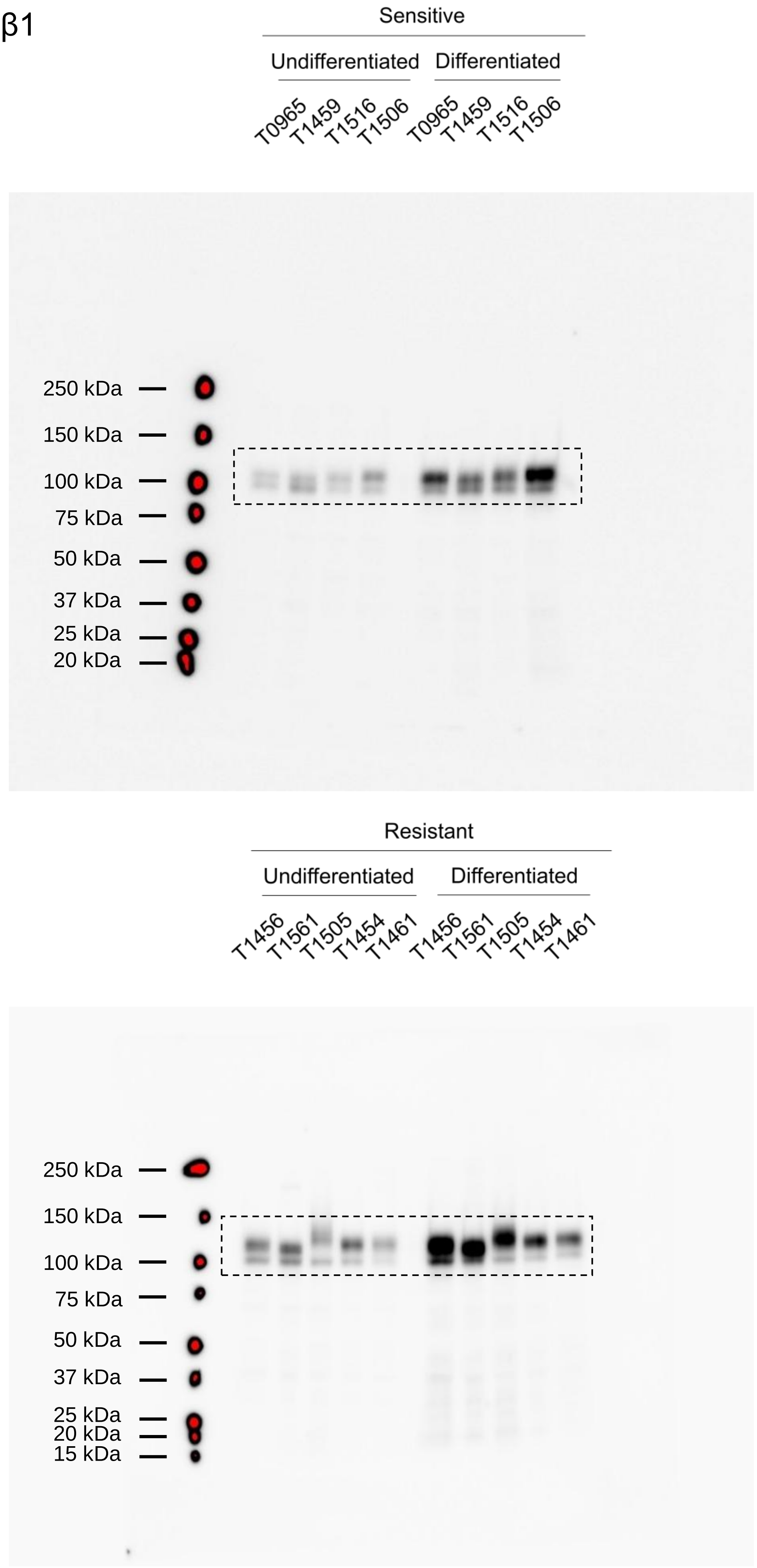

Supplementary Figure 4. *Full-length Western blot images for ITGβ1 corresponding to Fig. 2B. The cropped region is indicated and the relevant molecular weights are labeled.*

Fig 2B: SOX2

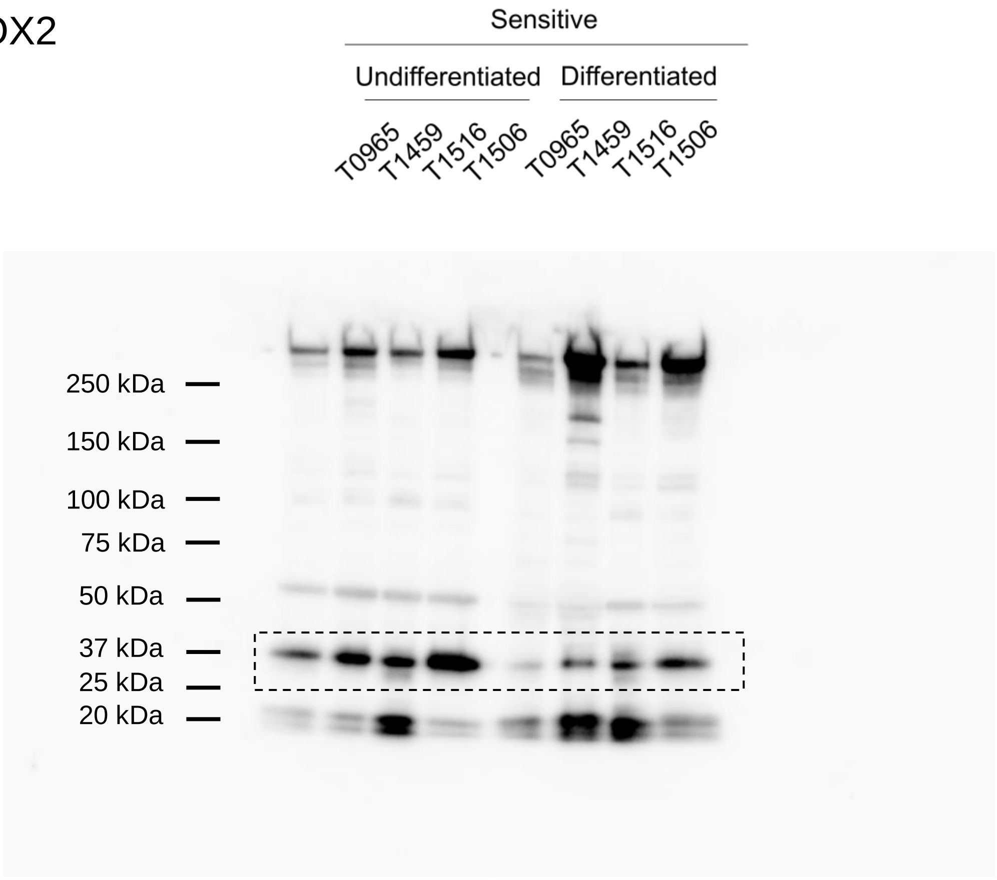

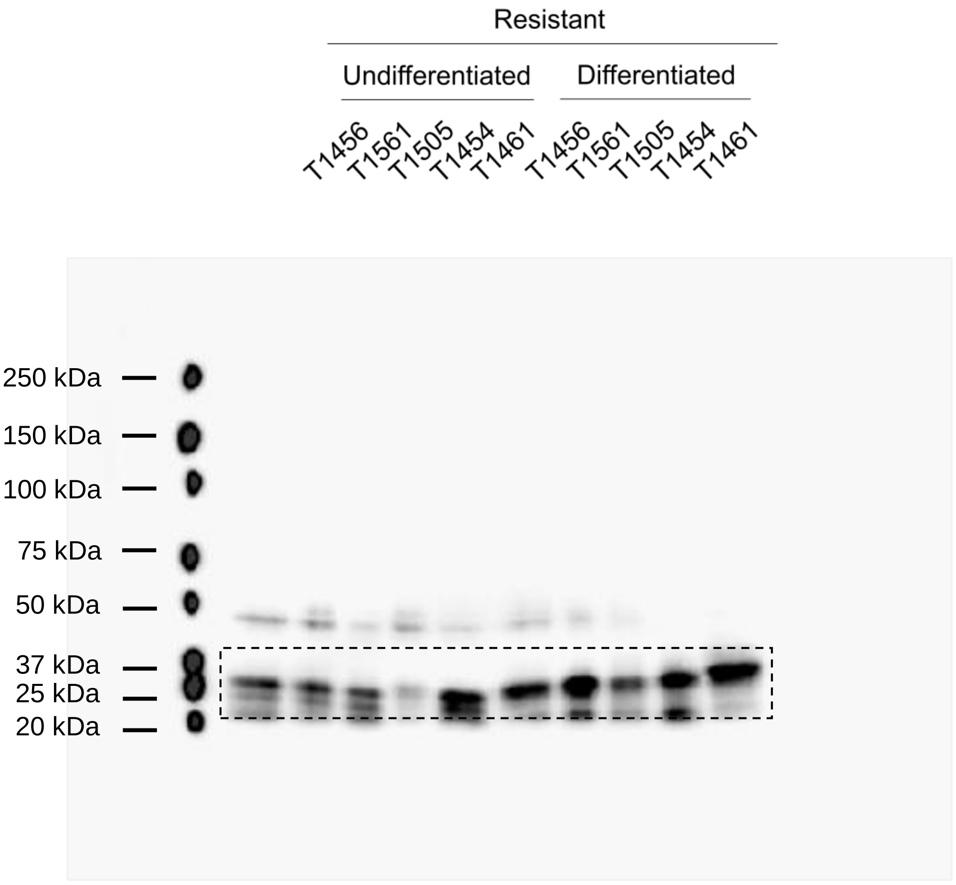

Supplementary Figure 5. *Full-length Western blot images for SOX2 corresponding to Fig. 2B. The cropped region is indicated and the relevant molecular weights are labeled.*

Fig 2B: β-actin

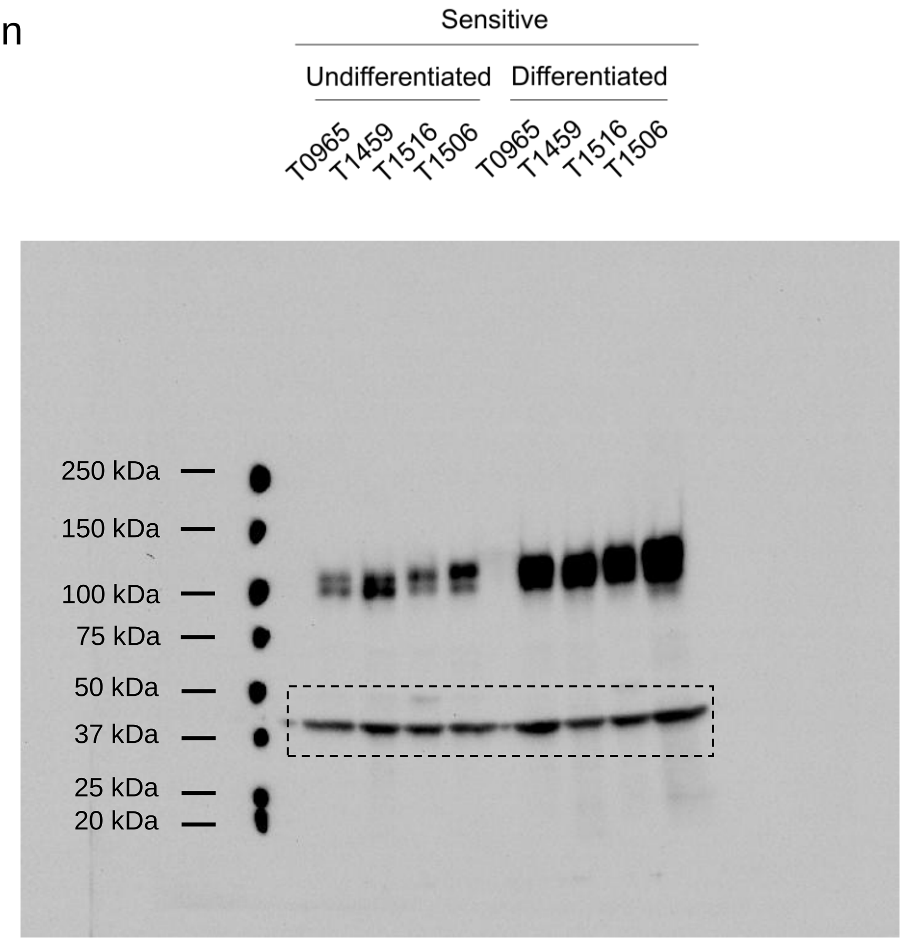

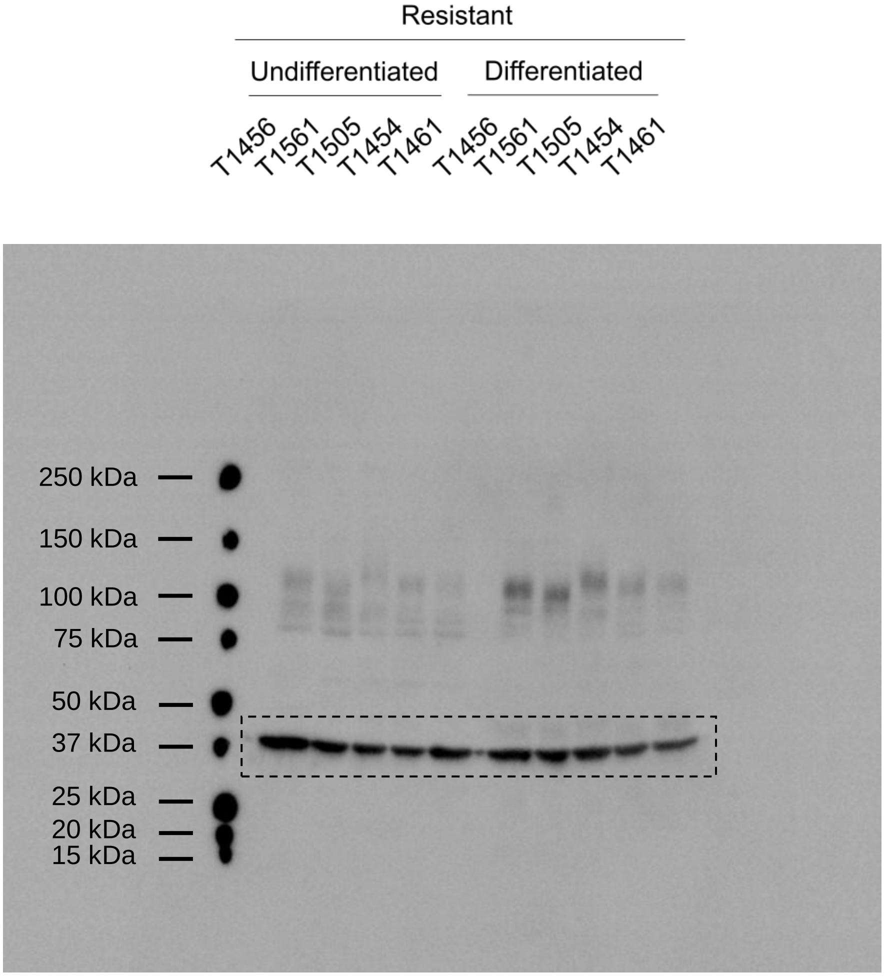

Supplementary Figure 6. *Full-length Western blot images for β-actin corresponding to Fig. 2B. The cropped region is indicated and the relevant molecular weights are labeled.*

Fig 2C: GFAP

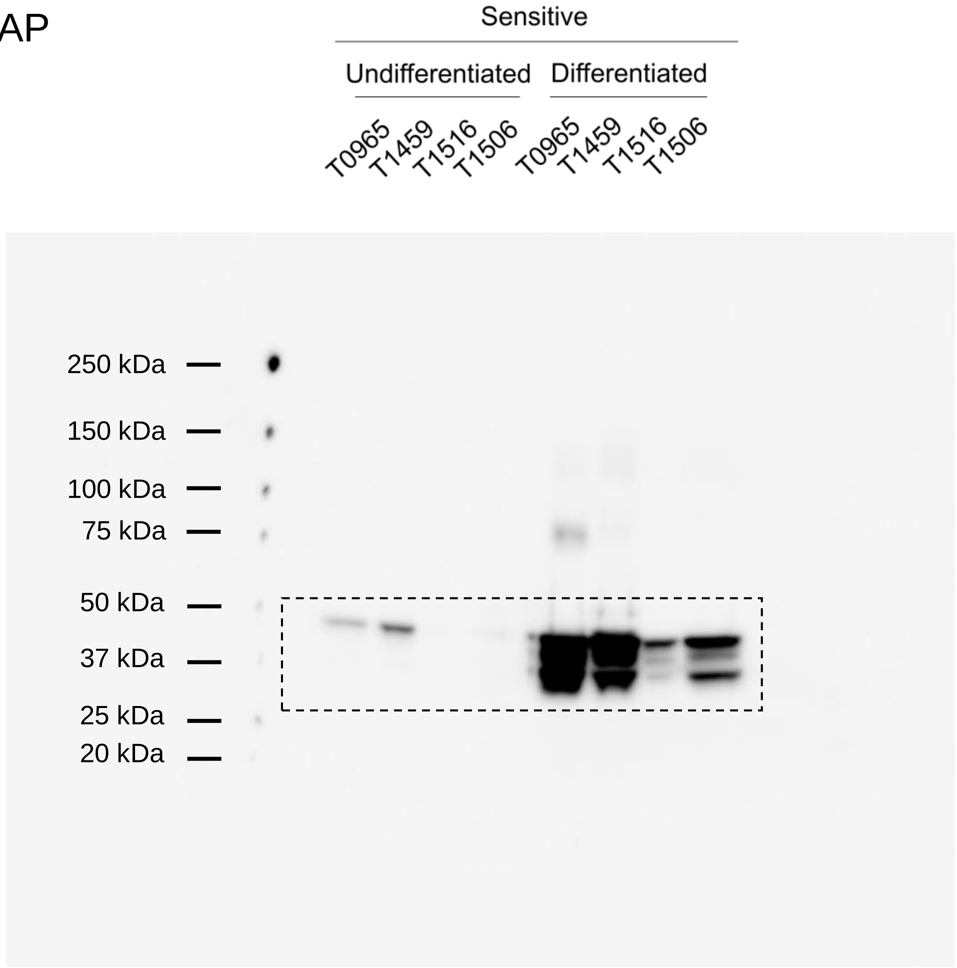

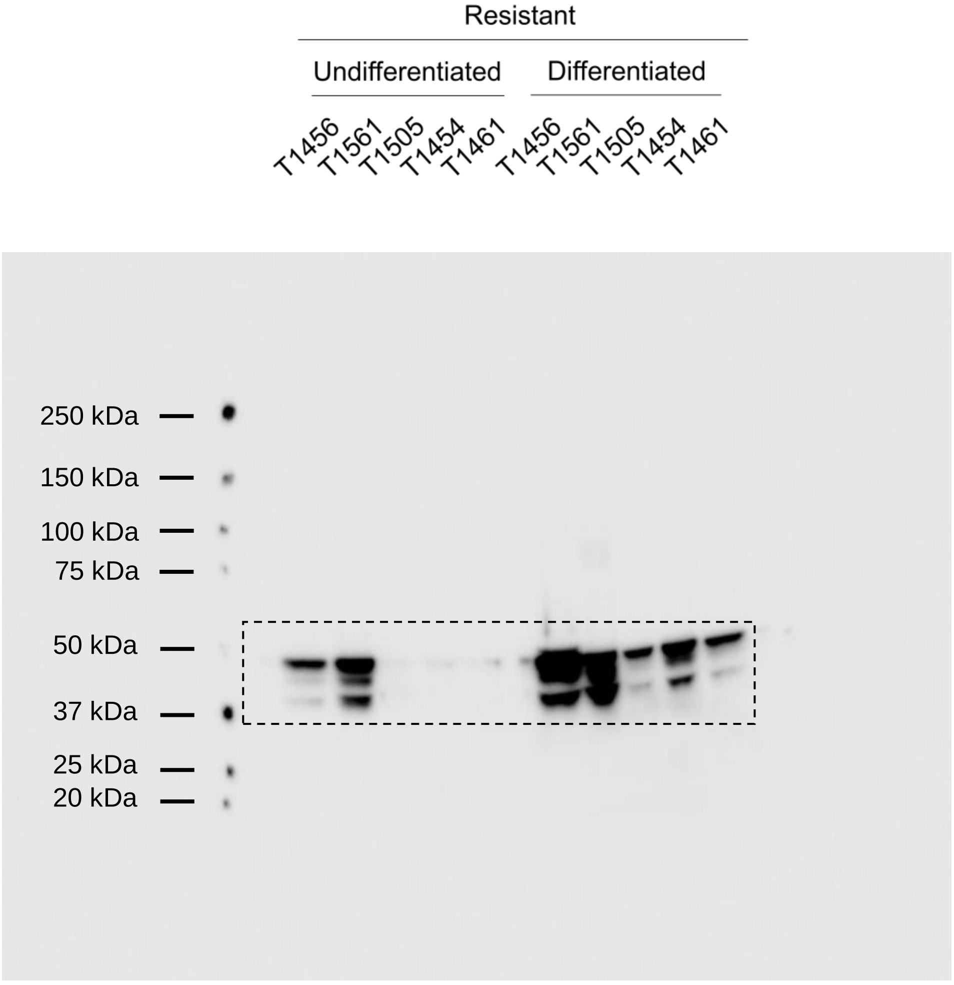

Supplementary Figure 7. *Full-length Western blot images for GFAP corresponding to Fig. 2C. The cropped region is indicated and the relevant molecular weights are labeled.*

Fig 2C: TUBB3

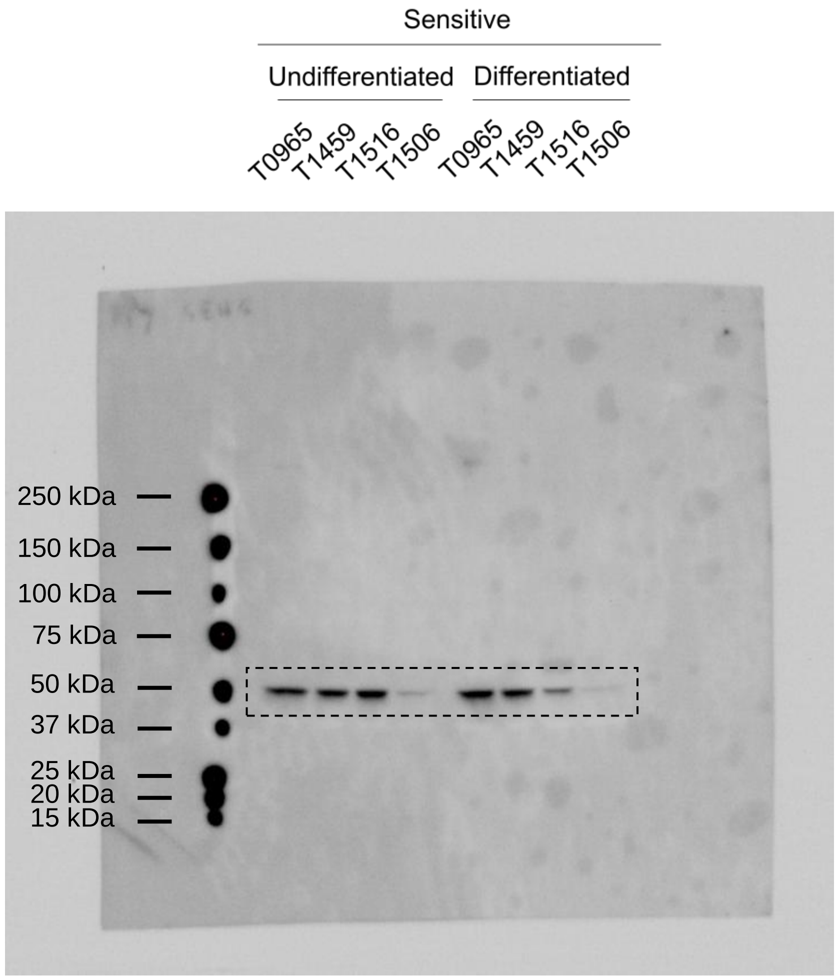

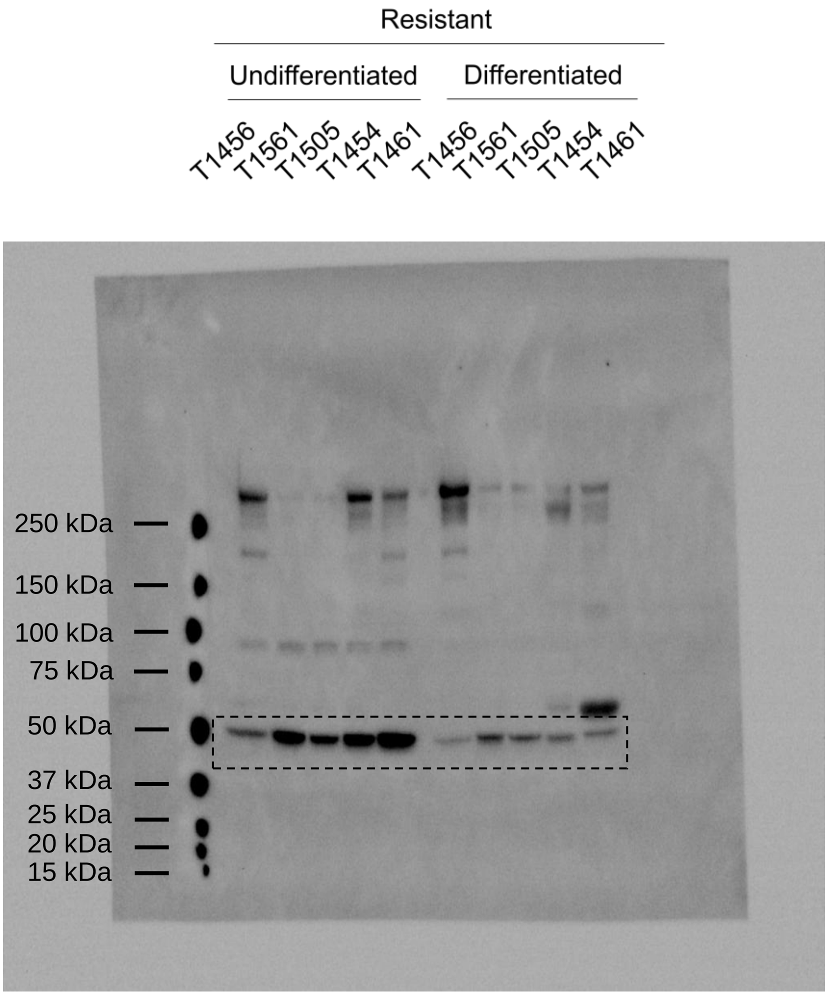

Supplementary Figure 8. *Full-length Western blot images for TUBB3 corresponding to Fig. 2C. The cropped region is indicated and the relevant molecular weights are labeled.*

Fig 2C: MAP2

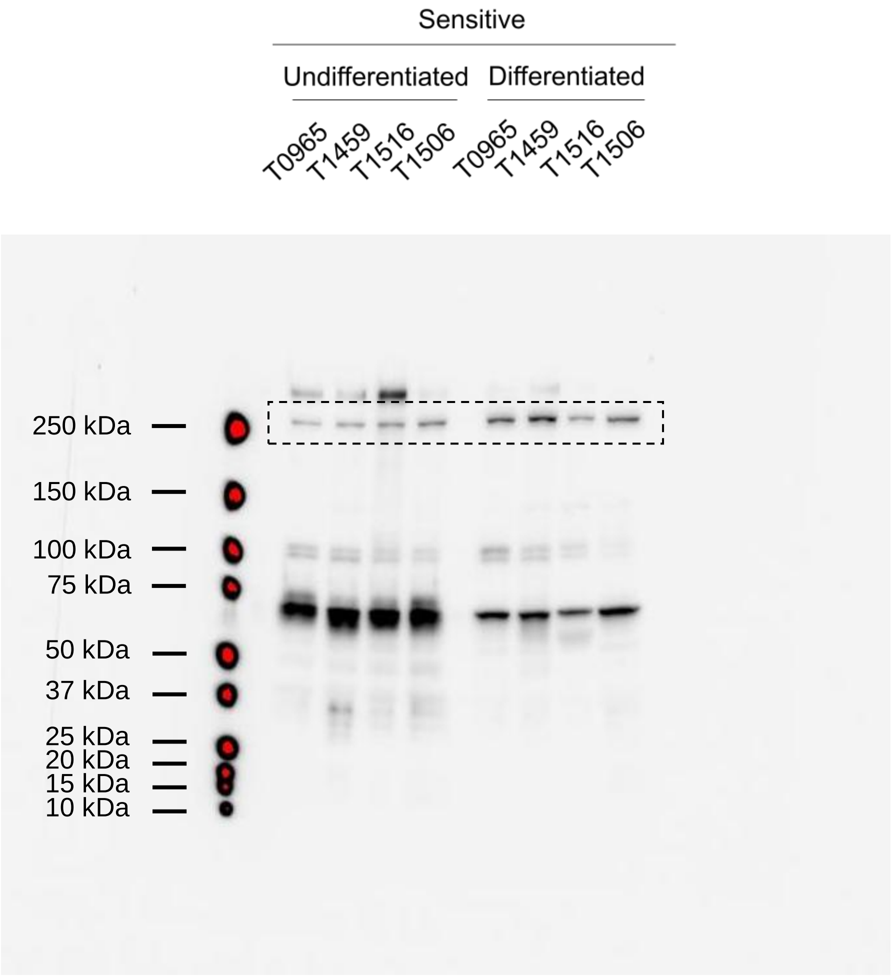

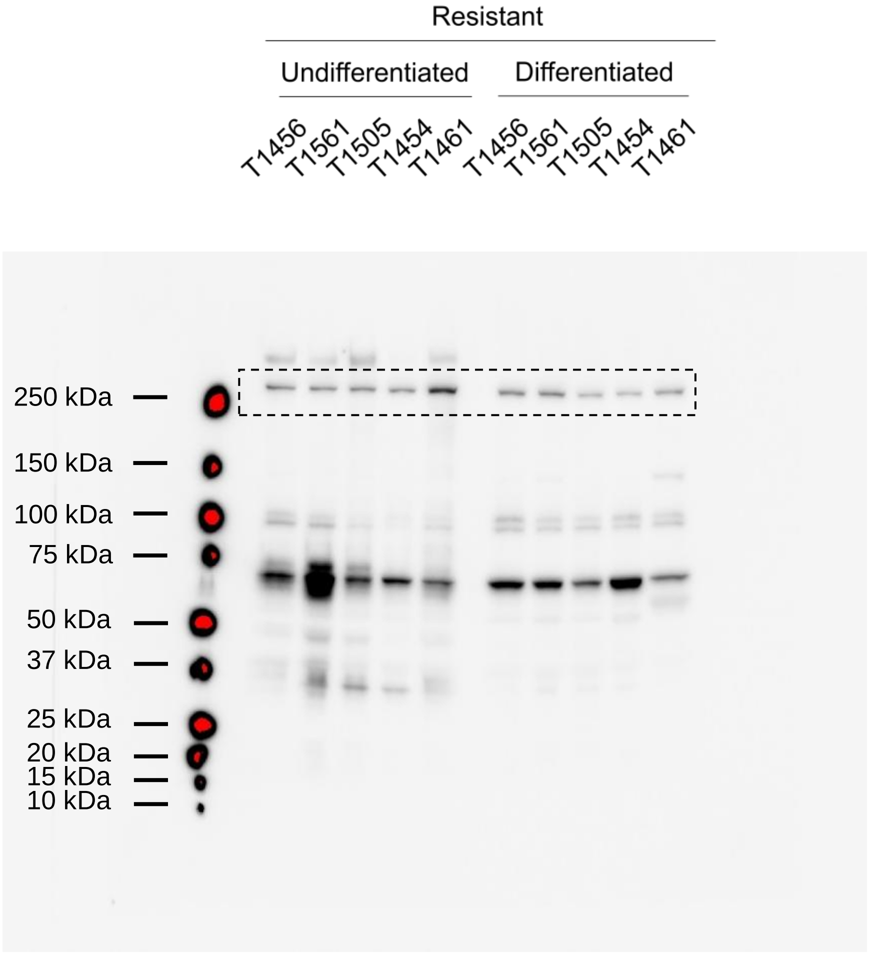

Supplementary Figure 9. *Full-length Western blot images for MAP2 corresponding to Fig. 2C. The cropped region is indicated and the relevant molecular weights are labeled.*

Fig 2C: CNPase

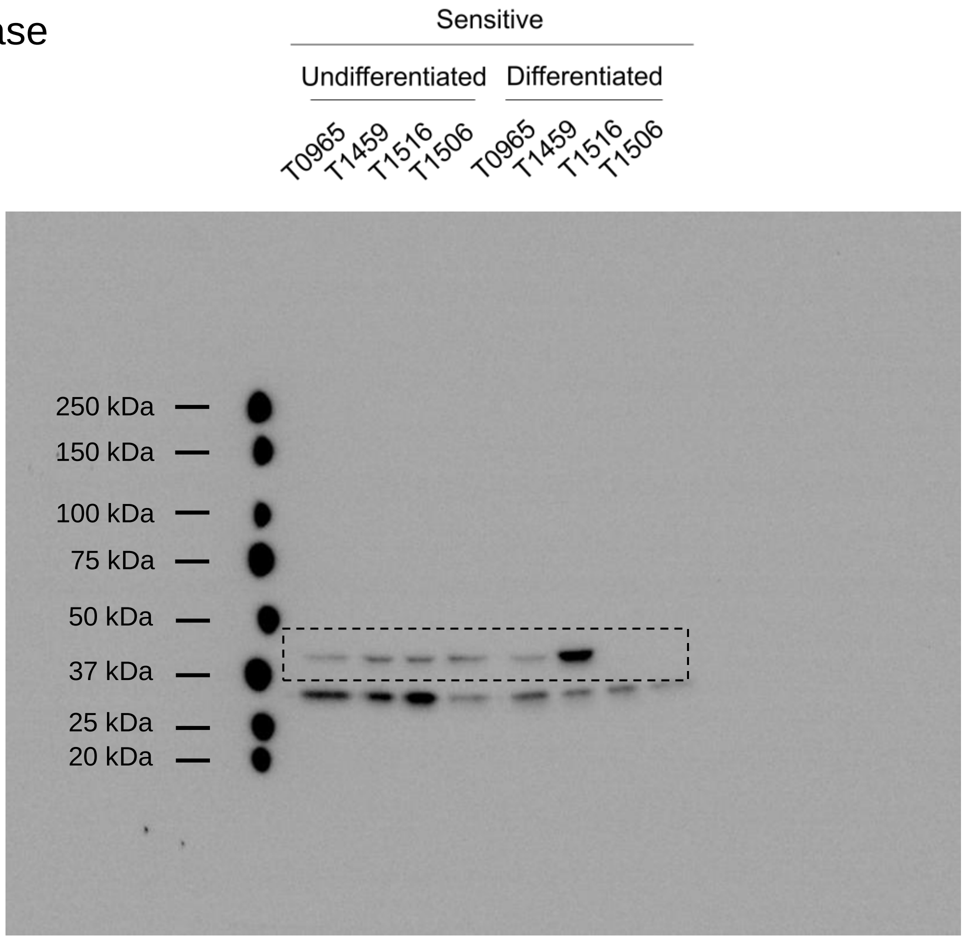

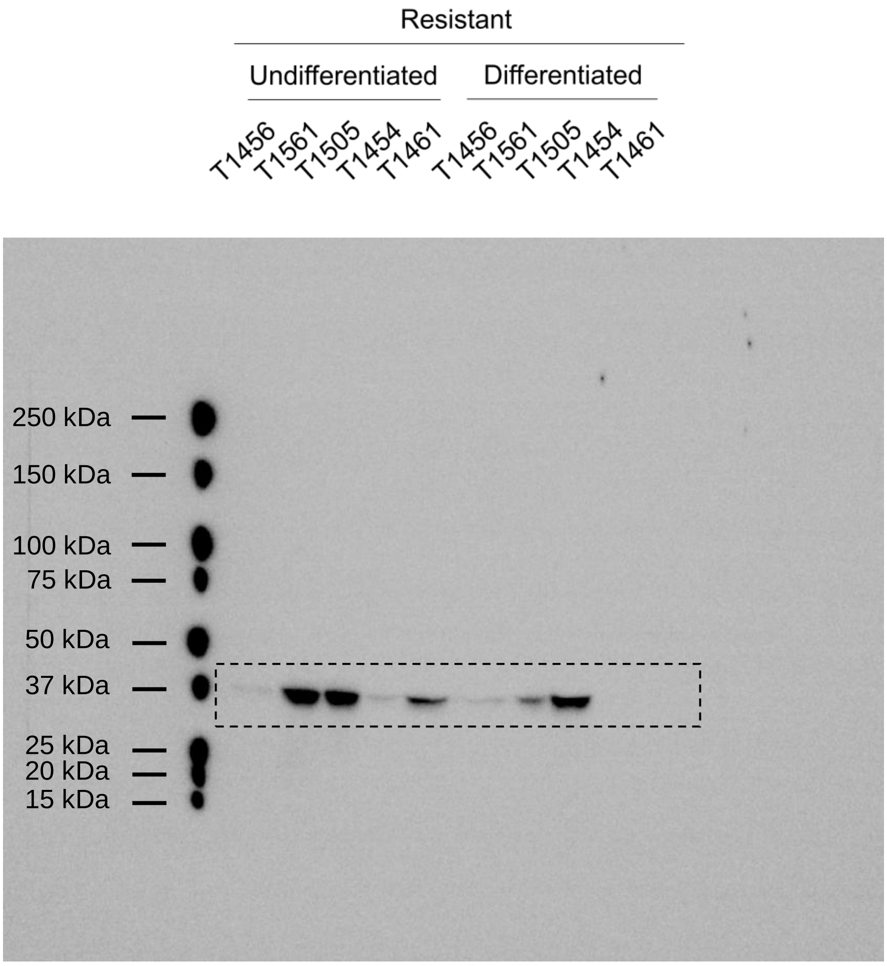

Supplementary Figure 10. *Full-length Western blot images for CNPase corresponding to Fig. 2C. The cropped region is indicated and the relevant molecular weights are labeled.*

Fig 2C: PCNA

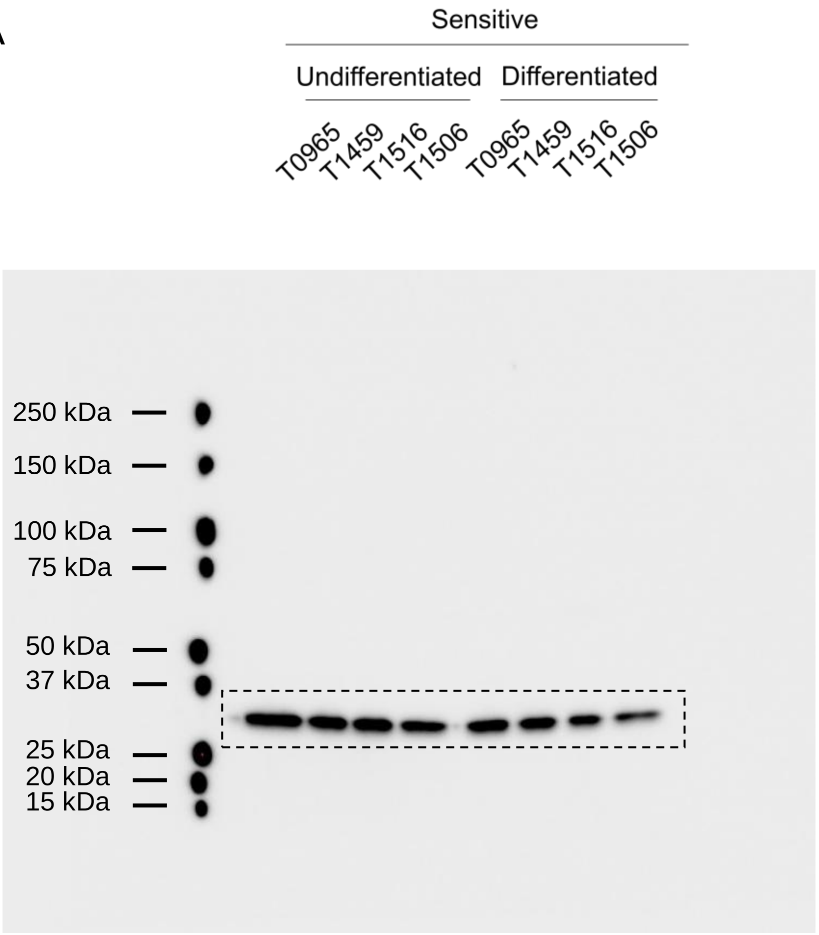

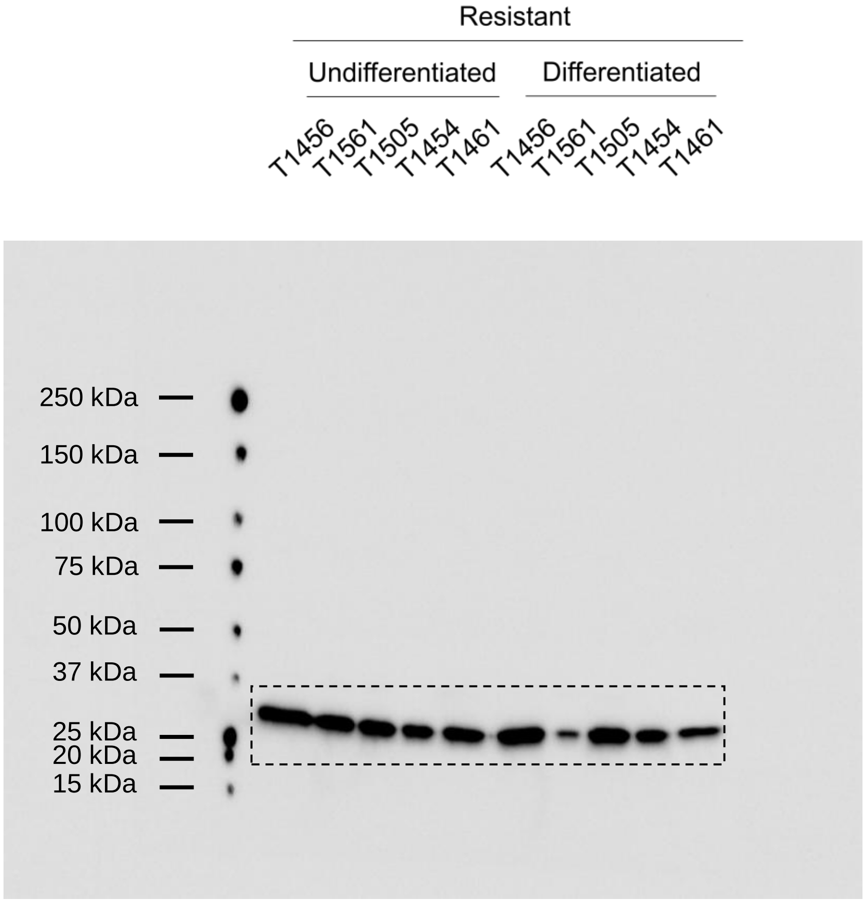

Supplementary Figure 11. *Full-length Western blot images for PCNA corresponding to Fig. 2C. The cropped region is indicated and the relevant molecular weights are labeled.*

Fig 2C: β-actin

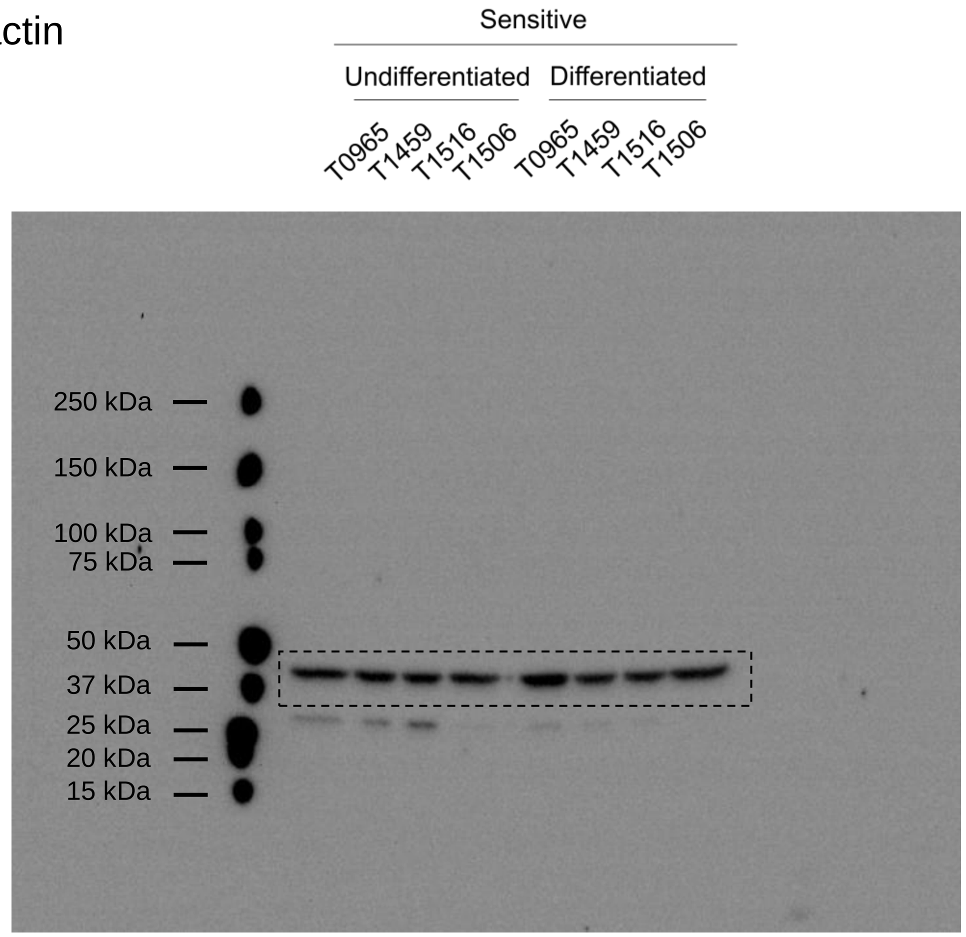

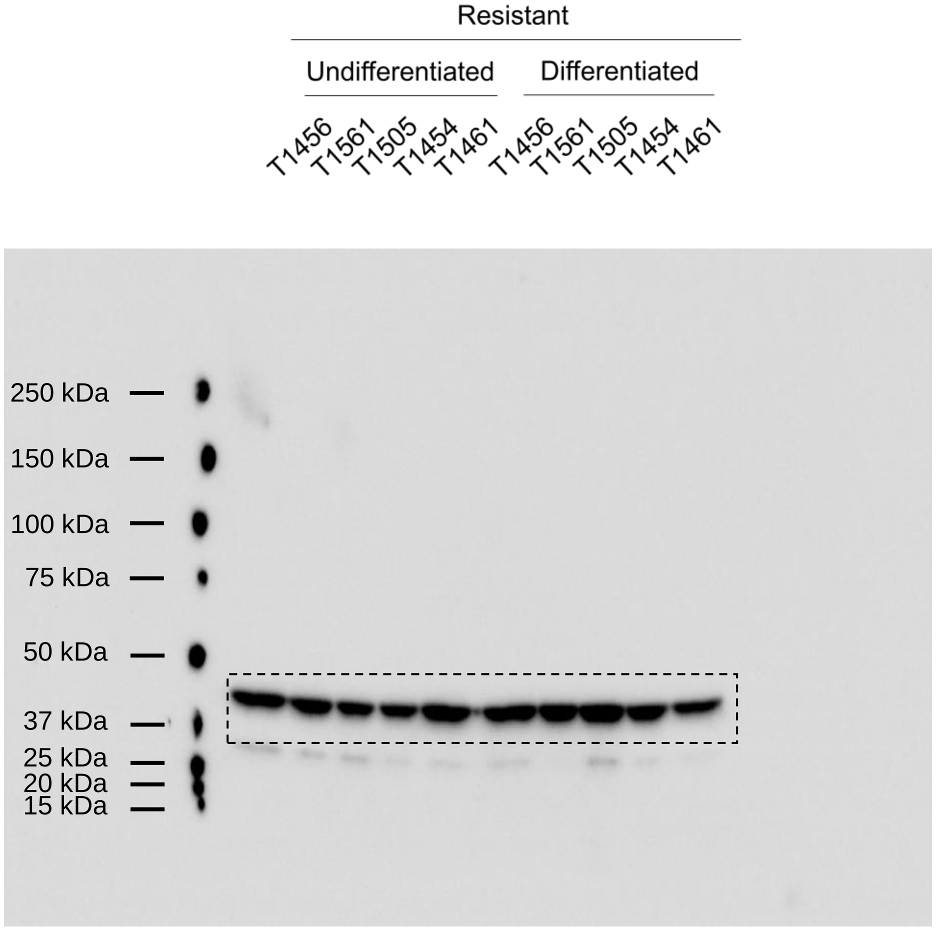

Supplementary Figure 12. *Full-length Western blot images for β-actin corresponding to Fig. 2C. The cropped region is indicated and the relevant molecular weights are labeled.*

Fig 4F: LOXL1

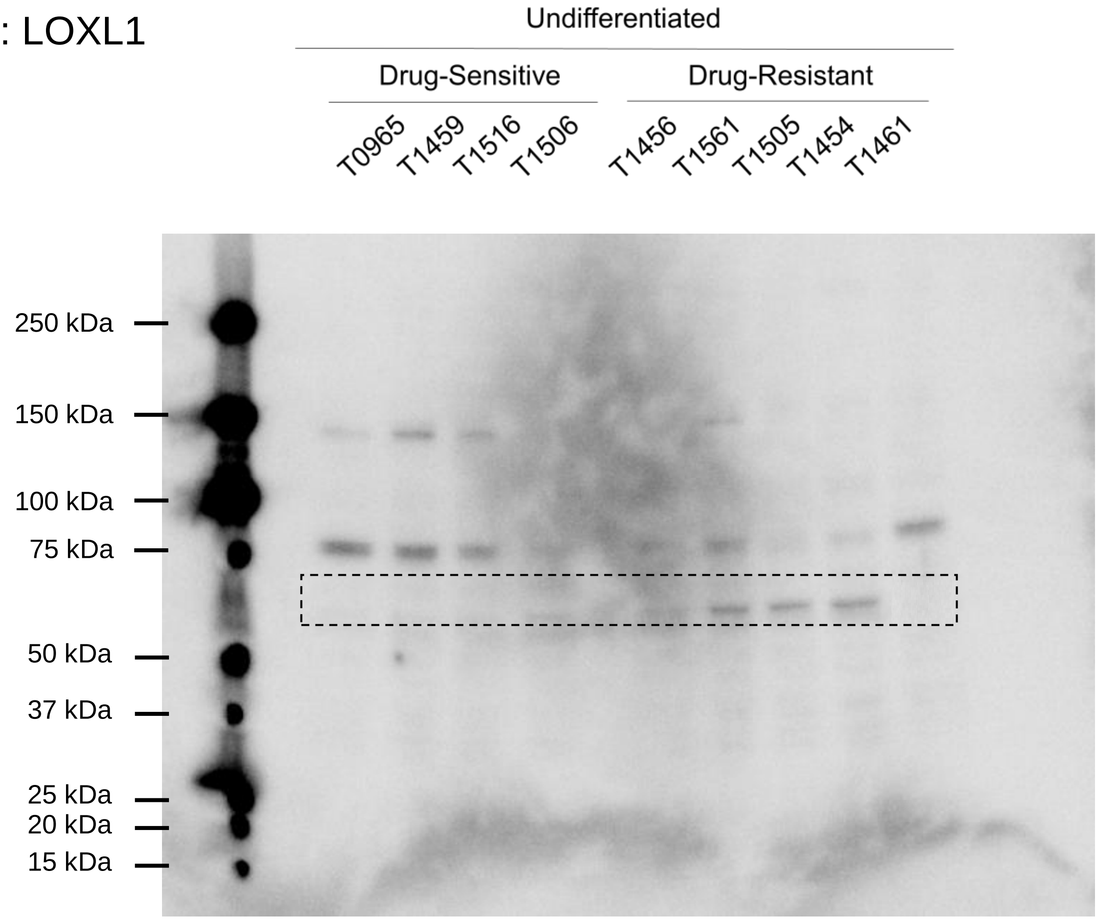

Fig 4F: COL25A1

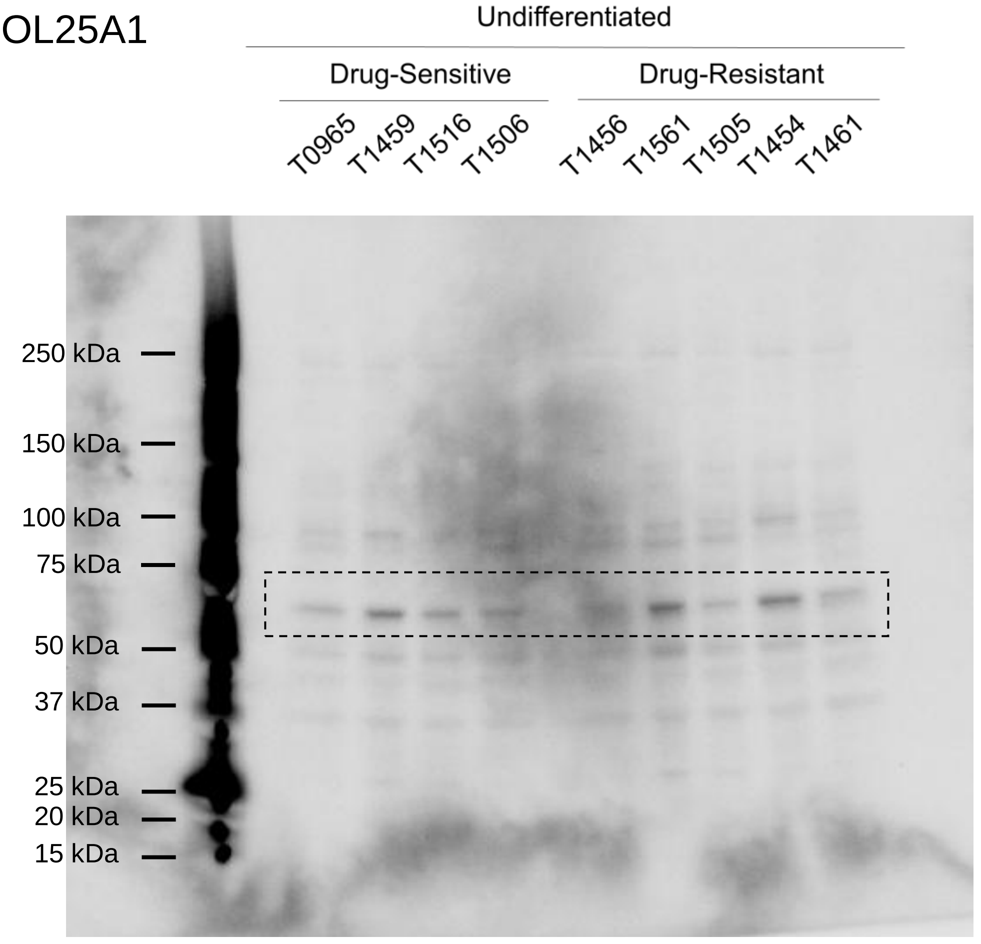

Supplementary Figure 13. *Full-length Western blot images for LOXL1 and COL25A1 corresponding to Fig. 4F. The cropped region is indicated and the relevant molecular weights are labeled.*

Fig 4F: ECM1

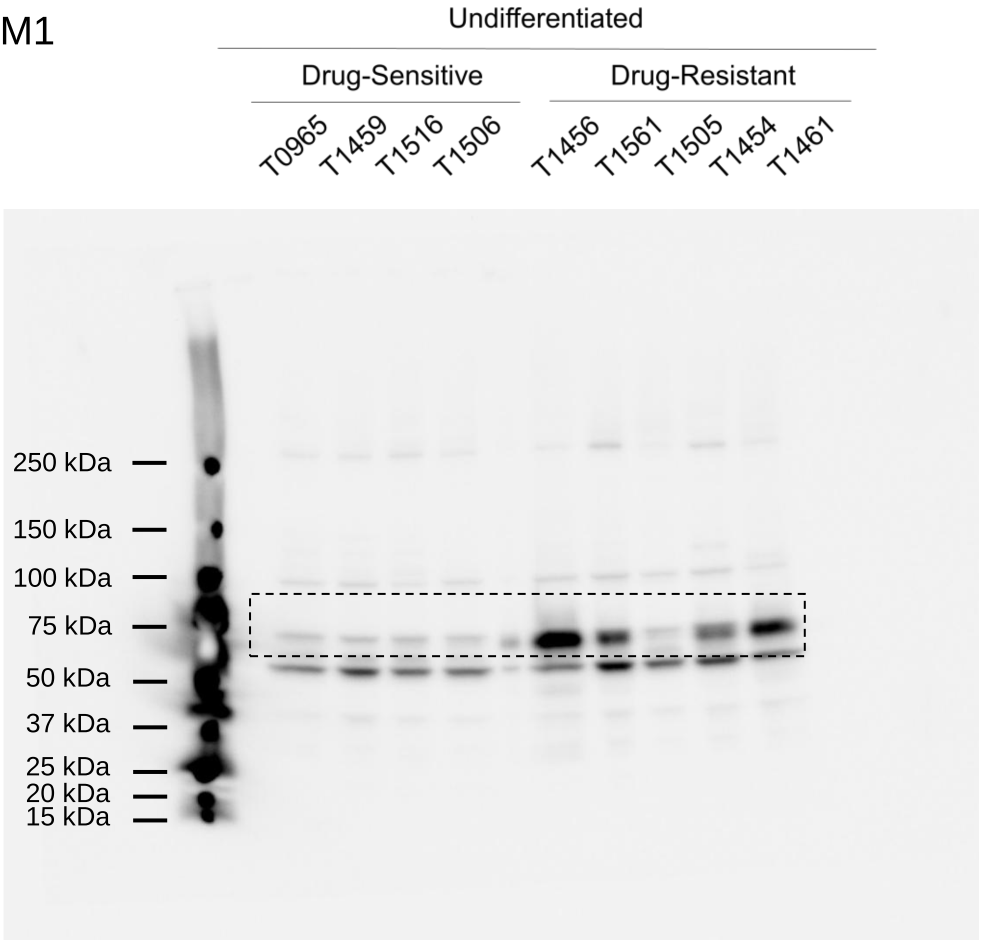

Fig 4F: β-actin

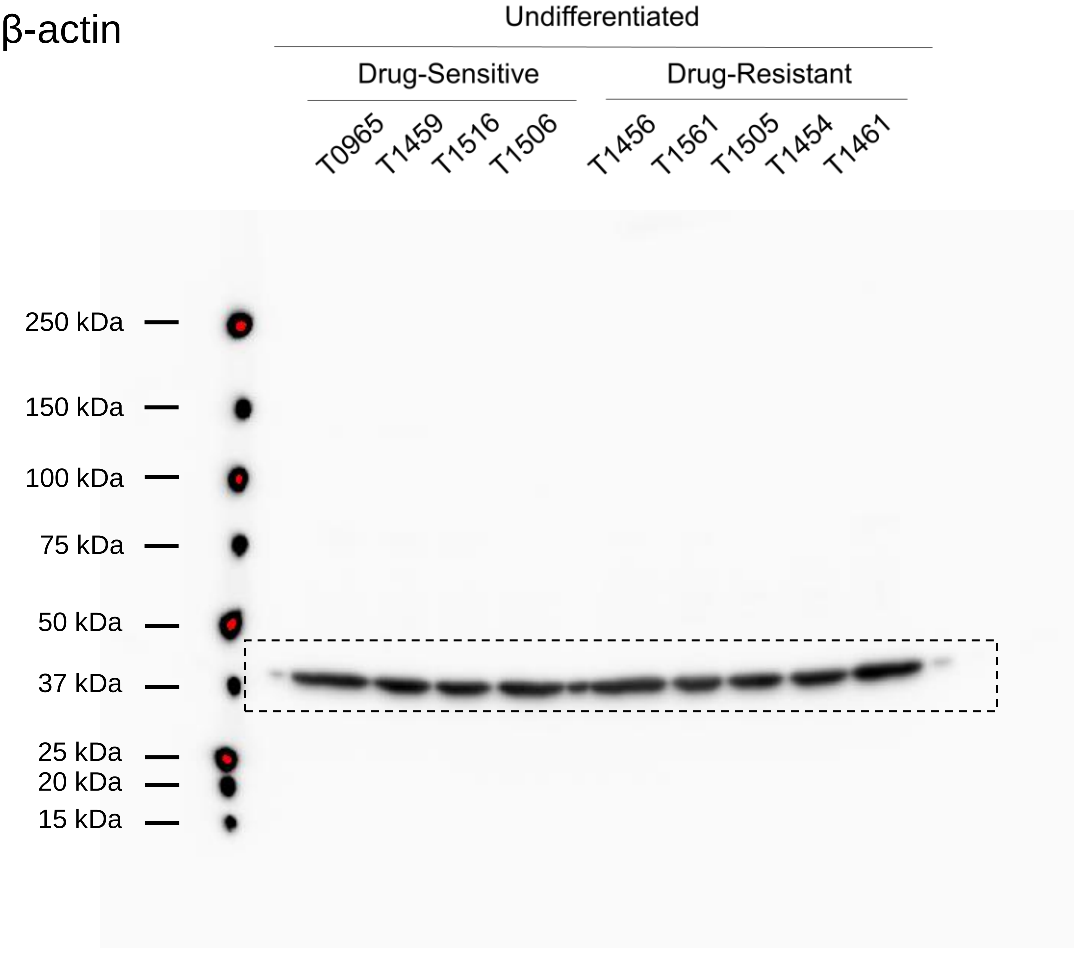

Supplementary Figure 14. *Full-length Western blot images for ECM1 and β-actin corresponding to Fig. 4F. The cropped region is indicated and the relevant molecular weights are labeled.*
