# Supplementary material for: Drug resistant glioblastoma stem cells exhibit enriched stemness signatures and share extracellular matrix overexpression
Source: BMC Cancer. 2025 Oct 27;25:1655. doi: 10.1186/s12885-025-15163-z (PMC12560458; doi:10.1186/s12885-025-15163-z)
Supplement: Supplementary file 8 — Additional file 8. Mutation profiles of drug-resistant and drug-sensitive GSC cultures. Somatic mutations identified by whole-exome sequencing, filtered for Tier I-III genes with an allele frequency > 5%. Mutations are classified by type, including missense, nonsense, splice region single-nucleotide variants (SNV), splice region deletions, and frameshift mutations, in each GSC culture. Samples with two distinct mutations are split diagonally, with each half representing a different mutation type. [file 12885_2025_15163_MOESM8_ESM.pdf]

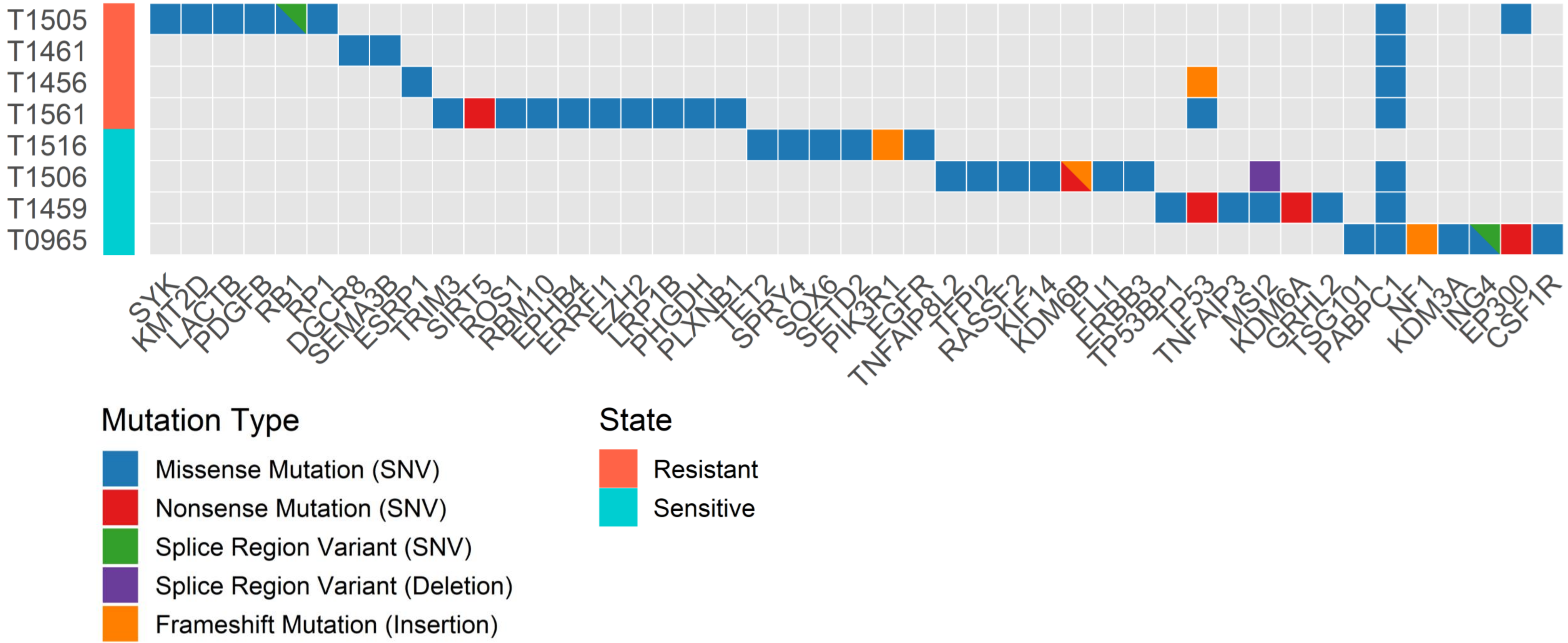

T1505
T1461
T1456
T1561
T1516
T1506
T1459
T0965
SYK
KMT2D
LACTB
PDGFB
RB1
RP1
DGCR8
SEMA3B
ESRP1
TRIM3
SIRT5
ROS1
RBM10
EPHB4
ERRFI1
EZH2
LRP1B
PHGDH
PLXNB1
TET2
SPRY4
SOX6
SETD2
PIK3R1
EGFR
TNFAIP8L2
TFPI2
RASSF2
KIF14
KDM6B
FLI1
ERBB3
TP53BP1
TP53
TNFAIP3
MSI2
KDM6A
GRHL2
TSG101
PABPC1
NF1
KDM3A
ING4
EP300
CSF1R
Mutation Type
Missense Mutation (SNV)
Nonsense Mutation (SNV)
Splice Region Variant (SNV)
Splice Region Variant (Deletion)
Frameshift Mutation (Insertion)
State
Resistant
Sensitive
